# A Digital Self-management Program (Help to Overcome Problems Effectively) for People Living With Cancer: Feasibility Randomized Controlled Trial

**DOI:** 10.2196/28322

**Published:** 2021-11-05

**Authors:** Hayley Wright, Faith Martin, Wendy Clyne, Cain C T Clark, Gabriela Matouskova, Michael McGillion, Andrew Turner

**Affiliations:** 1 Centre for Intelligent Healthcare Research Institute for Health and Wellbeing Coventry University Coventry United Kingdom; 2 National Institute for Health Research, Research Design Service South West Peninsula Medical School University of Plymouth Devon United Kingdom; 3 Hope For The Community, Community Interest Company The Enterprise Hub Coventry United Kingdom; 4 School of Nursing McMaster University Hamilton, ON Canada

**Keywords:** self-management, cancer, survivorship, digital, positive psychology

## Abstract

**Background:**

We present the results of a feasibility, randomized waitlist control group (CG) parallel design study with a 1:1 allocation ratio. Participants were randomized into an intervention group (IG) or a waitlist CG. The intervention was a 6-week digital self-management program, Help to Overcome Problems Effectively (HOPE), for people with cancer.

**Objective:**

This study aims to test the feasibility of a digitally delivered self-management program for people with cancer. This will inform the design of a definitive randomized controlled trial. In addition, a preliminary assessment of the impact of the HOPE program via secondary outcomes will be used to assess signals of efficacy in a trial context.

**Methods:**

Participants were drawn from an opportunity sample, referred by Macmillan Cancer Support, and were invited via email to participate in the study (N=61). Primary outcomes were rates of recruitment, retention, follow-up, completion and adherence, sample size and effect size estimation, and assessment of progression criteria for a definitive trial. Secondary outcomes were self-report measures of participants’ positive mental well-being, depression, anxiety, and patient activation (ie, confidence in managing their cancer). The intervention and data collection took place on the web.

**Results:**

The recruitment rate was 77% (47/61). A total of 41 participants completed the baseline questionnaires and were randomized to either the IG (n=21) or the waitlist CG (n=20). The retention rate (attending all program sessions) was greater than 50% (all: 21/41, 51%, IG: 10/21, 48%; and CG: 11/20, 55%). The follow-up rate (completing all questionnaires) was greater than 80% (all: 33/41, 80%; IG: 16/21, 76%; and CG: 17/20, 85%). The completion rate (attending ≥3 sessions and completing all questionnaires) was greater than 60% (all: 25/41, 61%; IG: 13/21, 62%; and CG: 12/20, 60%). Engagement data showed that participants viewed between half (5.1/10, 51%) and three-quarters (12.2/16, 76%) of the pages in each session.

**Conclusions:**

All progression criteria for a definitive trial were met, as supported by the primary outcome data. The IG showed improved postprogram scores on measures of positive mental well-being, depression, anxiety, and patient activation. A full-scale trial of the digital HOPE program for people with cancer will allow us to fully evaluate the efficacy of the intervention relative to a CG.

**Trial Registration:**

ISRCTN Registry ISRCTN79623250; http://www.isrctn.com/ISRCTN79623250

**International Registered Report Identifier (IRRID):**

RR2-10.2196/24264

## Introduction

### Background and Rationale

As of May 2021, the United Kingdom has seen 3 periods of national lockdown and widespread social and physical distancing measures implemented by the government in an attempt to curtail the spread of SARS-CoV-2. Such measures have resulted in significant reductions in the delivery of cancer services in the United Kingdom, as pressures on health services, lockdown demands, and the need to reduce face-to-face interactions have taken precedence [1]. Remote emotional support for these patients has recently been recommended by researchers [2] and cancer support specialists [3]. People with cancer are now experiencing increased health anxiety [4] owing to the risk of serious complications from the virus for people with cancer [5,6]. The need to implement alternative models of care during COVID-19, including more self-management support, has been noted by leading cancer care experts [7]. Moreover, before the COVID-19 pandemic, the UK National Health Service (NHS) had already called for greater emphasis on the delivery of digital, holistic, person-centered care for people with cancer [8]. Effective digital interventions have never been a more important aspect of care.

People with cancer are already known to face multiple challenges in terms of mental and physical health following primary treatment, including fatigue, pain, sexual problems, cognitive functioning, depression, anxiety, social isolation, and financial issues [9-13]. Furthermore, in the longer term, many patients with cancer experience negative impacts on their psychological well-being and mental health, including hypervigilance, anxiety, posttraumatic stress, and depression [14-20]. Research shows that 2 years after diagnosis, up to 20% of cancer survivors met the criteria for major depression and up to 40% met the criteria for an anxiety disorder [18-20]. As a result of the COVID-19 pandemic, people with cancer have reported a further decline in their mental and physical health [3].

Before the COVID-19 pandemic, there was a shortage of accessible self-management interventions, and there was an even greater need for digital interventions to comply with social distancing guidelines. Around a decade ago, we co-designed a face-to-face self-management program for survivors of all types of cancer [21,22]. People with cancer, oncologists, specialist cancer nurses, and representatives from a leading UK cancer charity (Macmillan Cancer Support; MCS) were involved in the co-design process. This led to the development of the *Help to Overcome Problems Effectively* (HOPE) program, which has been described in detail elsewhere [23,24]. The HOPE program aims to enhance well-being by fostering positive emotions and stimulating positive functioning. A parallel goal is to reduce depressive symptoms. The HOPE program is based on principles derived from positive psychology and focuses on positive experiences, strengths, and personal competencies rather than mental health problems such as anxiety and depression. It incorporates evidence-based exercises based on positive psychology, in addition to elements stemming from mindfulness, cognitive behavioral therapy, and problem-solving therapy. The HOPE program recognizes the common challenges and unmet needs across all types of cancer, including fatigue, fear of recurrence, and psychological distress [9-20]. The HOPE program was designed to provide support for these most common, typically overlapping needs in people living with most types of cancer. We regularly consult with MCS on their eHealth Needs Assessment data and review the most common needs indicated by people living with all types of cancer. The HOPE program provides general psychological and well-being support based on these needs and is open to all adult cancer survivors. The HOPE program differs from many other cancer self-management programs due to the focus on (1) positive psychology [25-27], (2) hope and gratitude [28] to improve well-being and coping, (3) co-created content, and (4) peer-facilitated delivery. The HOPE program is moderated by trained peer facilitators who are affected by cancer in some way. The facilitators received training from MCS and followed a delivery protocol. The facilitator’s role is to offer encouragement to participants and stimulate discussion in social networking forums by inviting participants to respond with comments to specific questions or respond to questions or comments posted by participants. Facilitators also monitor daily social networking posts for safety and report technical problems to the research team. The facilitators spent approximately 2 hours per session, supporting the participants. The in-person program was adapted for digital delivery (see [24] for full details of adaptation), using a user-centered, iterative approach [29]. A set of design requirements and a design brief were drawn up in consultation with end users and stakeholders. The initial digital version of HOPE went through a number of iterative testing sessions, with improvements made to usability after each iteration. These iterations were intended to develop a system that was usable and accepted by the intended user group to increase the likelihood of uptake and continued use and to ensure that the technology did not prove a barrier to engagement and participation. Initial evaluation has suggested positive effects on anxiety, depression, and positive well-being in people with cancer, with positive user feedback [23]. This suggests that a trial of the digital HOPE program might be viable and meaningful. A feasibility randomized controlled trial (RCT) study of the digital intervention was required to assess whether participants consent to be randomized and to test the feasibility of running a wait-list control study design of the HOPE program.

### Objectives

This study aims to test the feasibility of a digitally delivered self-management program for people with cancer. This will inform the design of a definitive RCT. In addition, a preliminary assessment of the impact of the HOPE program via secondary outcomes will be used to assess signals of efficacy in a trial context.

The planned primary outcomes (trial feasibility objectives) of the study were as follows: (1) recruitment rates for participation and for randomization; (2) retention and follow-up rates as the participants move through the trial; (3) adherence rates to study procedures, intervention attendance, and engagement; (4) sample size and effect size estimation for a definitive trial; and (5) progression criteria for a definitive trial.

The secondary outcomes related to participant well-being are measures of positive mental well-being, depression, anxiety, and confidence in self-managing cancer (patient activation), as indicated by scores on validated measures.

## Methods

The following sections were written in accordance with the 2016 CONSORT (Consolidated Standards of Reporting Trials) extension for pilot and feasibility trials [30].

### Trial Design

This study used a feasibility, randomized waitlist control group (CG) parallel design, with a 1:1 allocation ratio. Participants were randomized into an intervention group (IG) or a waitlist CG. The IG received access to the digital 6-week HOPE program immediately. The CG was placed on a waiting list for approximately 6 weeks, after which time they also received access to the same digital 6-week HOPE program. Key outcome measures were collected via web-based questionnaires at time 0 (T0; baseline) and time 1 (T1; 6 weeks postrandomization and postprogram for IG). We also sent the questionnaires to the CG only again after they had received the intervention (time 2; T2; postprogram for CG).

### Participants

The participants were referred by MCS, a leading UK cancer charity. They advertise the HOPE program through their social media networks, MCS websites, and word-of-mouth through specialist nurses.

Eligibility criteria for participants were as follows: (1) any cancer diagnosis, at any treatment stage; (2) adult (18 years or older), (3) located in the United Kingdom; (4) access to the internet and a device that allows them to engage with the intervention; and (5) fluent in English to be able to engage with all the material in the intervention.

All study data were collected on the web via questionnaires administered through the Qualtrics Survey Software (Qualtrics 2019; [31]).

### Intervention

All participants had access to the same digital HOPE program. The IG received access immediately, and the CG was granted access approximately 6 weeks later.

The HOPE program was delivered on the web. Full details of the digital HOPE program development, content, and weekly topics have been described elsewhere (see [23,24]), but we provide a brief overview here. All the HOPE program modules have the same structure and format, with a variety of components each week, focusing on a particular issue or a set of techniques, and ending with goal setting activity. The HOPE program is asynchronous, and content is released on a weekly basis at set times (eg, at midday every Monday) over the 6 weeks of the intervention. Forums and messaging facilities acted as a conduit for communication between participants and facilitators, and the program was moderated by trained peer facilitators. Table 1 provides an overview of the content and activities within each weekly module of the HOPE program.

**Table 1:** Examples of content, exercises, and activities within each weekly module of the Help to Overcome Problems Effectively program.

Session	Examples of content	Examples of exercises and activities (self-management tools)
Week 1 (Introduction or instilling hope)	Aims of the program User guide to navigating the platform and setting up a profile Introduction to self-management The benefits of positive emotions Video (Positive emotions for a flourishing life) The power of gratitude Personalized goal-setting Video (How to set achievable goals) Forum topic (Reasons for joining the program) Further resources and links (eg, videos, podcasts, and websites) to gratitude, positivity, and goal-setting	Interactive gratitude diary SMARTER^a^ goal-setting Assessment: positivity ratio test and positive and negative emotions test
Week 2 (Stress management)	Understanding stress Managing stress Videos (How to manage stress and how to make stress your friend) Coping with unhelpful thinking patterns Mindfulness for stress management and meditation Self-compassion and acceptance Video (How to be kind to yourself) Forum topic (How do you deal with cancer-related stress?) Further resources and links (eg, videos, podcasts, and websites) to self-compassion, mindfulness, and stress management	Interactive gratitude diary SMARTER goal-setting and goal feedback Guided relaxation and meditation exercise (podcasts) How to cope with unhelpful thoughts (worksheet)
Week 3 (Managing fatigue)	Understanding the boom and bust cycle Using the 3 Ps (prioritizing, planning, and pacing) for managing fatigue Video (Tips for managing fatigue) Sleeping better; podcast: Tips to improve sleep Forum topic (Coping with fatigue) Further resources and links (eg, videos, podcasts, and websites) to sleeping better	Interactive gratitude diary SMARTER goal-setting and goal feedback Fatigue and pacing diaries (worksheets) Quiz (What are the main challenges faced by cancer survivors?)
Week 4 (Body image and communication)	Body image Video (Body image and cancer) Sexuality and intimacy Video (Cancer as a passport to emotional intimacy) Communication skills and tips for talking with the health care team and family Forum topic: experiences of coping with body changes and experiences of communicating with the health care team Further resources and links (eg, videos, podcasts, and websites) to sexuality, intimacy, and relationships	Interactive gratitude diary SMARTER goal-setting and goal feedback
Week 5 (Physical activity and fear of recurrence)	Coping with fear of recurrence Videos (Moving forward while being worried about cancer returning and the regrets of those who are dying) Hopes and dreams for the future Video: Before I die project The benefits of physical activity Video (Tips for becoming and staying active) Forum topic (Concerns about cancer coming back) Further resources and links (eg, videos, podcasts, and websites) to managing concerns about cancer coming back and getting more active	Interactive gratitude diary SMARTER goal-setting and goal feedback
Week 6 (Character strengths and happiness)	Understanding how using your strengths can lead to a more fulfilling life Video (The science of character strengths) Tips for authentic happiness; managing setbacks and keeping going Forum topic (Learning from the program) Further resources and links (eg, videos, podcasts, and websites) to MCS^b^ web-based communities and happiness resources	Interactive gratitude diary SMARTER goal-setting and goal feedback Assessment (positivity ratio test and positive and negative emotions test and character strengths) Quiz (What contributes to happiness?)

^a^SMARTER: SMARTER is an acronym used by many organizations for goal-setting, and stands for specific, measurable, achievable, relevant, time-bound, enjoyable, and reward.

^b^MCS: Macmillan Cancer Support.

### Primary Outcomes

The primary outcome measures for this feasibility RCT were as follows:

#### Recruitment Rates

Recruitment rates for participation and randomization were collected primarily through Qualtrics at the start of the trial. All eligible participants identified by MCS were sent a link to the Qualtrics study survey. Recruitment rates were then calculated from the following: (1) providing consent and (2) completing baseline questionnaires. Direct email from participants indicating refusal or declining to participate in the study indicated a refusal. These participants were still offered access to the HOPE program but did not participate in any further data collection.

#### Retention, Follow-up, and Completion Rates

The participant retention rate was calculated as the percentage of participants attending all 6 program sessions. Studies show that a median of 56% of participants complete the full program in digital interventions for mental well-being [32,33]. As high rates of nonuse attrition [34] are common and of concern in digitally delivered interventions, and because of uncertainties relating to the COVID-19 pandemic, we set a more conservative target of 50% of participants completing all 6 sessions of the intervention.

Follow-up was calculated as the percentage of participants who completed all web-based study questionnaires. Participants who were lost to follow-up were identified through Qualtrics as those who did not complete the postprogram questionnaires at the end of the intervention period. It is possible that these participants may still have attended some portions or the entire HOPE program, despite not completing questionnaires.

If participants attended at least half of the intervention (3 sessions) [32] and completed the study questionnaires, they were classified as intervention completers. Studies show a nonlinear relationship between the time spent on an intervention, the number of sessions completed, and outcomes [32]. The amount of use needed to obtain desired outcomes varies across groups, and individuals may stop using the intervention once personal goals are achieved [35]. Therefore, we set a more pragmatic target for our primary outcome measure of *completion rate* of at least 3 sessions attended, and completion of all study questionnaires.

#### Adherence and Engagement Measures

The intervention platform collects user engagement data, such as the number of pages viewed in each session and the number of goals set that assists the moderators with participant engagement and experience. We measured the mean percentage of pages viewed per session, and the number of posts or comments a participant made for key activities (gratitude, setting goals, goal feedback, liking posts, and comments posted).

#### Sample Size and Effect Size Estimation for Future Definitive Trial

To inform the sample size estimation for a future definitive trial, we calculated the SDs of key continuous secondary outcomes at baseline. To estimate potential effect sizes for a primary outcome in a future definitive trial from pre- to postprogram, we calculated the difference between the mean difference pre- and postprogram for the IG and CG and divided by the pooled SD at baseline [36].

#### Progression Criteria

There is little guidance available for determining progression criteria for exploratory studies, including feasibility trials [37]. Following good practice, our progression criteria were discussed with Patient and Public Involvement representatives [38] and within the trial project group. Our 3 progression criteria reflect specific uncertainties regarding the feasibility of a larger definitive trial. The web-based HOPE program has not previously been delivered in an RCT study context, so two of our progression criteria tested the willingness of people to participate in a trial (recruitment and questionnaire completion rate). Full intervention completion (now labeled as “retention” within the manuscript) is the most frequently reported metric for adherence to web-based interventions [39], which, through discussion, was set as our third progression criteria.

To inform the progression to a definitive trial, we compared our results to the progression criteria set *a priori* as follows: (1) recruitment rate >70% of eligible participants consented, (2) questionnaire completion rate >70% of participants completing T1 questionnaires, and (3) retention rate >50% of participants attending all 6 HOPE program sessions.

### Secondary Outcomes

Sociodemographic and health data were collected at T0. Participants were asked to provide the following information via a web-based questionnaire: gender, age, ethnicity, marital status, highest level of education, employment and occupation, and details about their cancer diagnosis and other medical conditions.

Participants completed a set of validated questionnaires at T0 (baseline), T1 (6 weeks postrandomization), and T2 (postprogram for CG only). Postprogram (T1 and T2) questionnaires were made available to participants the week after the intervention ended and remained available for a further 4 weeks. The positive mental well-being, depression, anxiety, and patient activation measures are detailed below.

The Warwick Edinburgh Mental Well-being Scale (WEMWBS) [40] is a validated scale of 14 positively worded feelings and thoughts used to assess mental well-being within the adult population. The scale includes measures of positive affect, satisfying interpersonal relationships and positive functioning, for example, *Below are some statements about feelings and thoughts. Please tick the box that best describes your experience of each over the last 2 weeks*...(1) “I have been feeling optimistic about the future,” (2) “I have been thinking clearly,” and (3) “I have been feeling loved.” Participants rated each of the 14 items on a scale of 1 to 5 (1=none of the time, 2=rarely, 3=some of the time, 4=often, 5=all of the time), providing a total positive mental well-being score ranging from 14 to 70, with higher scores representing greater positive mental well-being. The WEMWBS had good internal consistency (Cronbach α=.91). A change of 3 or more was seen as clinically meaningful change [41].

The 9-item Patient Health Questionnaire (PHQ-9) [42] is a validated 9-item measure, which assesses the frequency of experience of the symptoms of depression. For example, *Over the past 2 weeks, how often have you been bothered by any of the following problems*...(1) *little interest or pleasure in doing things*; (2) *feeling down, depressed, or hopeless*; and (3) *poor appetite or overeating*.

Responses to each of the 9 items ranged from 0 to 3 (0=not at all, 1=several days, 2=more than half the days, 3=nearly every day), leading to a summed score between 0 and 27, with higher scores indicating greater severity of depression. The PHQ-9 has good internal consistency (α=.89). Scores of 10 or more are presumed to be above the clinical range; thus, participants scoring 10 are categorized as depressed for the purpose of this study.

The 7-item Generalized Anxiety Disorder scale (GAD-7) [43] is a validated 7-item scale measuring symptoms of generalized anxiety disorder, for example, *Over the past 2 weeks, how often have you been bothered by the following problems*...(1) *feeling nervous, anxious or on edge*; (2) *trouble relaxing*; and (3) *becoming easily annoyed or irritable*. Responses to all 7 items ranged from 0 to 3 (0=not at all, 1=several days, 2=more than half the days, 3=nearly every day), providing a total score of 0 to 21, with higher scores indicating greater anxiety. GAD-7 has good internal consistency (Cronbach =.92). Scores of 8 or more are presumed to be above the clinical range; therefore, participants scoring 8 are categorized as having anxiety for the purpose of this study.

The Patient Activation Measure [44] is a validated, licensed tool with good internal consistency (Cronbach =.81), which has been extensively tested with reviewed findings from a large number of studies. It helps to measure the spectrum of knowledge, skills, and confidence in patients and captures the extent to which people feel engaged and confident in taking care of their condition. Individuals are asked to complete a short survey, and based on their responses, they receive a Patient Activation Measure score (between 0 and 100). The resulting score places the individual at 1 of 4 levels of activation, each of which reveals insight into a range of health-related characteristics, including behaviors and outcomes. The 4 levels of activation are as follows:

1. Level 1 (scores ≤47.0): Individuals tend to be passive and feel overwhelmed by managing their own health. They may not understand their roles in the care process.
2. Level 2 (scores 47.1-55.1): Individuals may lack the knowledge and confidence to manage their health.
3. Level 3 (scores 55.2-67.0): Individuals appear to be taking action but may still lack the confidence and skill to support their behaviors.
4. Level 4 (scores 67.1): Individuals have adopted many of the behaviors needed to support their health but may not be able to maintain them in the face of life stressors.

### Sample Size for This Study

All study participants were drawn from an opportunity sample (N=61), provided by MCS, of eligible candidates who expressed an interest in taking part in the HOPE program. An arbitrary sample size of n=40 was deemed adequate for this feasibility study, informed by similar studies in this area, with sample sizes ranging from 10 to 20 in each arm [45]. All potential study participants were emailed a link to the study website hosted by Qualtrics, where they were asked to read the digital Participant Information Sheet, read and agree to the statements on the digital consent form, and complete the digital T0 questionnaire before randomization.

### Randomization

#### Sequence Generation

All participants who provided informed consent and completed the T0 questionnaires were randomized into the IG or CG using a 1:1 ratio via the randomization function within the Qualtrics Survey Software.

#### Allocation Concealment Mechanism

Participants were informed on completion of the T0 questionnaires, via a notification in Qualtrics, whether they had been randomized to the IG (in this case, starting in May 2020), or the CG (in this case, starting in June 2020). The research team remained unaware of participant allocation until group contact lists were created at the next data collection point (ie, T1).

#### Implementation

Participants were allocated to the IG or CG via the randomization function in Qualtrics. Participants were then emailed with a link to the HOPE program starting in the following week (IG), or a message to say that they would be emailed a link to the HOPE program (CG) in approximately 6 weeks’ time.

### Blinding

Owing to the nature of the study design, it was not possible to blind the participants to their group allocation. However, statistical analyses of study data were conducted blind to participant allocation where possible (eg, IG and CG were labeled *A* and *B* arbitrarily).

### Analytical Methods

Quantitative data were analyzed descriptively using IBM SPSS 26 (IBM Corporation, released 2019). Initial analyses involved tabulated and graphical summaries of primary and secondary outcomes for each randomized group using means and variance, including CIs and SDs, and number and percentages for categorical variables to describe the full range of data at baseline and postprogram. An intention-to-treat (ITT) analysis was carried out, where missing data were rectified using the last observation carried forward (LOCF) [46]. In line with CONSORT (Consolidated Standards of Reporting Trials) guidelines [47], a per-protocol (PP) analysis was also performed on secondary outcome data from intervention completers and is reported in the *Ancillary* *Analyses* section.

The study was not powered to perform inferential statistical analyses, and so to signal efficacy, we report pre- and postprogram mean differences and CIs for scores on key secondary outcome measures for the IG and CG.

### Protocol

The feasibility trial protocol has been registered and published (International Registered Report Identifier IRRID: DERR1-10.2196/24264) [24].

## Results

### Participant Flow

**Figure 1:**
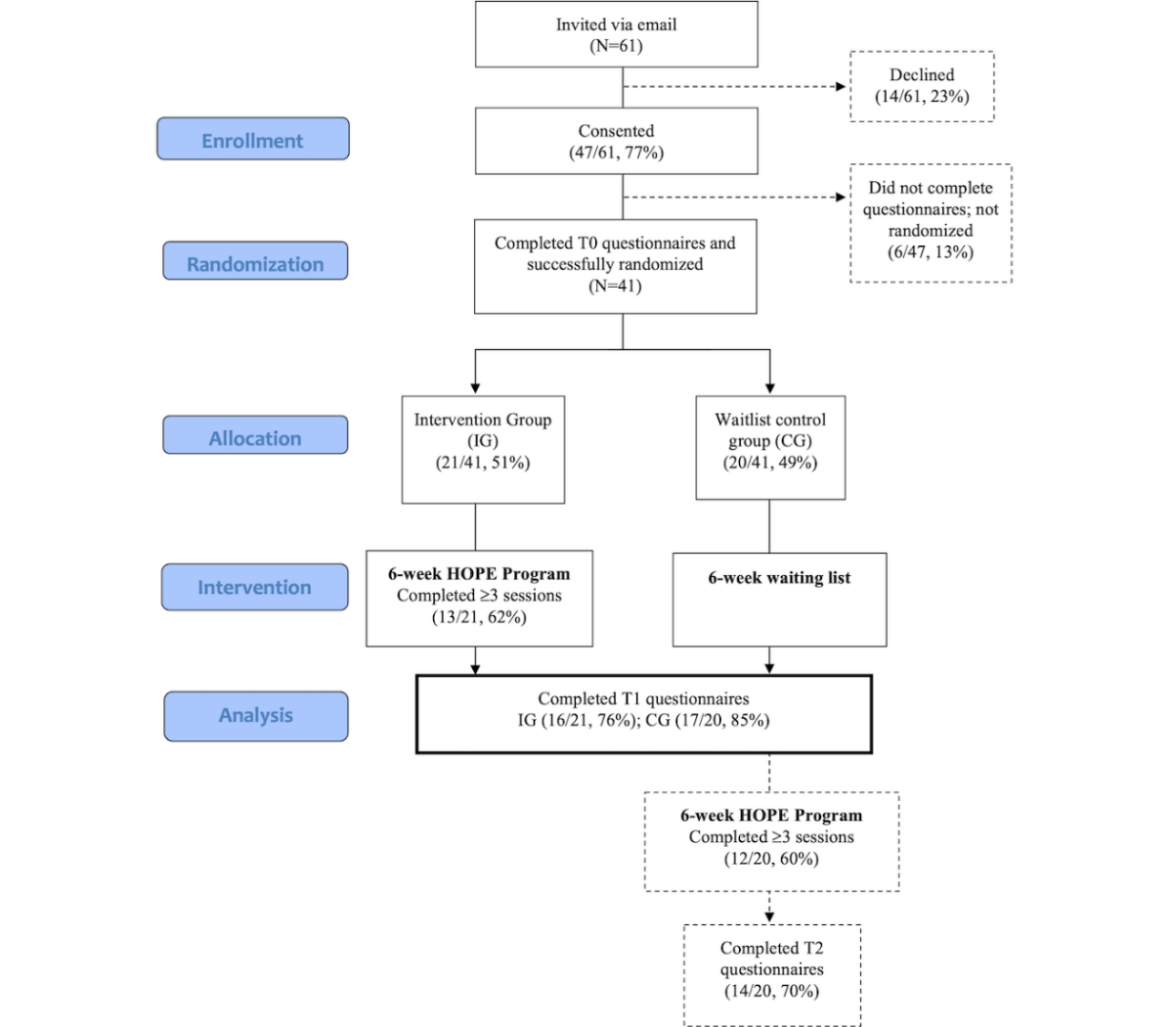
Participant flow through the study. HOPE: Help to Overcome Problems Effectively.

Figure 1 provides the details of the participant flow through the study.

### Recruitment

Recruitment started on April 30, 2020, and ended on May 5, 2020. Data collection started on April 30, 2020, for T0 baseline questionnaires and finished on September 2, 2020, for T2 follow-up questionnaires (CG only), which was 4 weeks after the end of the intervention for the CG as specified in the trial protocol.

### Baseline Data

Sociodemographic and health information collected at baseline (T0) for the whole group and by treatment group are presented in Table 2. The sample consisted mostly of White participants (36/41, 87%), women (32/41, 78%) with an average age of 54.3 years. More than half of the sample (24/41, 58%) had postschool qualifications. Most participants were married or living with their partner (30/41, 73%), and more than half were employed (21/41, 51%), with just under half reporting that they had to reduce their working hours because of their cancer diagnosis (20/41, 49%). This variable was the most disproportionate across the trial arms, with more than twice as many reports of cutting work hours in the IG (14/21, 67%) than in the CG (6/20, 30%) The most commonly reported type of cancer was breast cancer (17/41, 41%), with less than half of participants still undergoing treatment (17/41, 41%). Participants may have reported more than one type of cancer; therefore, the sum of the group percentages may be >100%.

**Table 2:** Baseline characteristics for the whole sample and by trial arm.

Variable			All (N=41)		IG^a^ (n=21)		CG^b^ (n=20)
Age, mean (SD)			54.3 (11)		52.6 (11)		56.2 (12)
Female, n (%)			32 (78)		17 (81)		15 (75)
White ethnicity, n (%)			36 (88)		19 (90)		17 (85)
Married or living with partner, n (%)			30 (73)		17 (81)		13 (65)
Employed, n (%)			21 (51)		11 (52)		10 (50)
Cut work hours due to cancer, n (%)			20 (49)		14 (67)		6 (30)
Possessed postschool qualifications, n (%)			24 (58)		12 (57)		12 (60)
Still undergoing treatment for cancer, n (%)			17 (41)		9 (43)		8 (40)
**Cancer type, n (%)**							
	Breast	17 (41)		8 (38)		9 (45)	
	Gynecological	4 (10)		0 (0)		4 (20)	
	Prostate	2 (5)		0 (0)		2 (10)	
	Lung	3 (7)		2 (9)		1 (5)	
	Colorectal	3 (7)		3 (14)		0 (0)	
	Gastrointestinal	2 (5)		0 (0)		2 (10)	
	Bladder or kidney	1 (2)		0 (0)		1 (5)	
	Head or neck	4 (19)		2 (9)		2 (5)	
	Other	9 (22)		8 (38)		1 (5)	

^a^IG: intervention group.

^b^CG: control group.

### Numbers Analyzed

The total number of participants enrolled in the study was 41, with 21 in the IG and 20 in the CG. All participants completed baseline (T0) questionnaires, and missing data in T1 and T2 questionnaires were populated with the LOCF method for ITT analysis. Therefore, the entire sample was included in the ITT analysis (ITT, N=41; IG, n=21; CG, n=20). The numbers for the PP analysis are detailed in the *Ancillary* *Analyses* section.

### Outcomes and Estimation

We describe the results for each primary and secondary outcome. Primary outcome measures pertaining to recruitment, questionnaire, and intervention completion rates are presented in Figure 1, Table 3, and Table 4. Secondary outcome measures are presented in Table 5 for the whole group (n=41) and each trial arm (IG, n=21; CG, n=20).

**Table 3:** Number of participants who attended none, some, or all of the Help to Overcome Problems Effectively Program sessions, for the whole group and each trial arm.

Number of sessions attended	All (N=41), n (%)	IG^a^ (n=21), n (%)	CG^b^ (n=20), n (%)
0	5 (12)	2 (9)	3 (15)
1	5 (12)	3 (14)	2 (10)
2	5 (12)	3 (14)	2 (10)
3	2 (5)	1 (5)	1 (5)
4	1 (2)	0 (0)	1 (5)
5	2 (5)	2 (9)	0 (0)
6	21 (51)	10 (47)	11 (55)

^a^IG: intervention group.

^b^CG: control group.

**Table 4:** An overview of engagement and adherence with the Help to Overcome Problems Effectively Program, for the whole group and by trial arm.

Engagement measure	All (N=41), n (%)	All (n=41), mean (SD)	IG^a^ (n=21), n (%)	IG (n=21), mean (SD)	CG^b^ (n=20), n (%)	CG (n=20), mean (SD)
Mean pages viewed in session 1 (range 0-16)	12.2 (76)	12.2 (6.4)	12.2 (76)	12.2 (6.2)	12.2 (76)	12.2 (6.7)
Mean pages viewed in session 2 (range 0-13)	8.5 (65)	8.5 (6.0)	8.0 (61)	8.0 (6.1)	9.1 (70)	9.1 (6.0)
Mean pages viewed in session 3 (range 0-17)	10.3 (60)	10.3 (8.0)	9.7 (57)	9.7 (8.0)	10.9 (64)	10.9 (8.2)
Mean pages viewed in session 4 (range 0-14)	7.5 (53)	7.5 (6.8)	7.0 (50)	7.0 (7.0)	8.1 (58)	8.1 (6.9)
Mean pages viewed in session 5 (range 0-16)	8.2 (51)	8.2 (8.0)	7.7 (48)	7.7 (8.0)	8.8 (55)	8.8 (8.2)
Mean pages viewed in session 6 (range 0-10)	5.1 (51)	5.1 (4.6)	5.0 (50)	5.0 (4.4)	5.3 (53)	5.3 (4.9)
Gratitude entries across whole program (range 0-9)	N/A^c^	1.5 (1.9)	N/A	1.3 (1.3)	N/A	1.7 (2.4)
Goals set across whole program (range 0-8)	N/A	2.1 (2.1)	N/A	1.8 (1.8)	N/A	2.4 (2.4)
Goal feedback given across whole program (range 0-5)	N/A	0.5 (1.1)	N/A	0.4 (1.0)	N/A	0.7 (1.2)
Likes given across whole program (range 0-56)	N/A	6.8 (12.2)	N/A	3.8 (6.6)	N/A	10.0 (15.8)
Comments posted across whole program (range 0-35)	N/A	6.8 (9.9)	N/A	5.8 (9.2)	N/A	7.9 (10.7)

^a^IG: intervention group.

^b^CG: control group.

^c^N/A: not applicable.

**Table 5:** All scores, intention-to-treat, on secondary outcome measures for intervention control (IG) and control group (CG), and change in scores (T1-T0), and mean difference in changes scores (IG-CG).

Secondary outcome measure	IG (n=21)				CG (n=20)				Difference in change scores Δ IG-CG, mean difference (95% CI)
	T0, mean (SD)	T1^a^, mean (SD)	Postprogram change Δ (T1-T0), mean difference (95% CI)	T0, mean (SD)		T1^b^, mean (SD)	Control change Δ (T1-T0), mean difference (95% CI)		
WEMWBS^c^	43.3 (9.6)	46.3 (11.7)	3.0 (−0.2 to 6.2)	43.4 (12.4)		45.1 (11.8)	1.7 (−1.6 to 5.0)	1.3 (−3.1 to 5.7)	
PHQ-9^d^	10.0 (5.4)	8.1 (5.7)	−1.8 (−3.3 to −0.4)	9.2 (6.3)		9.3 (6.5)	0.1 (−2.0 to 2.2)	−1.9 (−4.4 to 0.6)	
GAD-7^e^	8.8 (5.6)	7.6 (5.6)	−1.2 (−3.0 to 0.6)	9.0 (6.6)		7.4 (4.8)	−1.6 (−3.7 to 0.5)	0.4 (−2.2 to 3.1)	
PAM^f^	58.8 (17.2)	60.8 (17.2)	2.0 (0.1 to 3.9)	61.3 (15.3)		59.1 (14.2)	−2.2 (−6.6 to 2.2)	4.2 (−0.3 to 8.7)	

^a^LOCF: last observation carried forward, n=5.

^b^LOCF: last observation carried forward, n=3.

^c^WEMWBS: Warwick-Edinburgh Mental Well-being Scale.

^d^PHQ-9: 9-item Patient Health Questionnaire.

^e^GAD-7: 7-item Generalized Anxiety Disorder scale.

^f^PAM: Patient Activation Measure.

#### Primary Outcomes

##### Recruitment rates

The recruitment rate for this feasibility study was 77% (47/61). MCS referred to 61 participants for the study, and 77% of participants (47/61) provided digital informed consent. Only participants who (1) consented, (2) completed baseline questionnaires, and (3) were randomized, were included in this feasibility study (n=41). Six participants provided informed consent but did not complete the T0 questionnaire; therefore, they were not randomized (6/47, 13%). The study participants who completed the baseline questionnaires (n=41) were randomized to either the IG (n=21) or the CG (n=20) groups.

##### Retention, Follow-up, and Completion Rates

The retention rate across the sample was 51%, with more than half of the participants attending all 6 program sessions (whole group: 21/41, 51%; IG: 10/21, 47%; CG: 11/20, 55%).

Across the whole group, there was a follow-up rate of 80% (33/41 participants completed T1 questionnaires). Across the trial arms, the follow-up rate was 76% (16/21) in the IG and 85% (17/20) in the CG.

The completion rates (3 sessions and T1 questionnaire) for the whole group were 61% (25/41), 62% (13/21) in the IG, and 60% (12/20) in the CG.

##### Adherence and engagement measures

Table 4 shows the selection of the engagement data collected by the intervention platform. The mean number of pages viewed per session generally decreased as the program progressed and ranged from 12.2/16 (76%) in session 1 to 5.1/10 (51%) in session 6, across the whole group. The mean pages viewed in each session were consistent across the entire group and both trial arms for sessions 1 and 6. The mean number of pages viewed was slightly higher for the CG than for sessions 2 to 5. Furthermore, the CG tended to set slightly more goals, give more likes, and post more comments than the IG, on average, across the course of intervention. There was a negligible difference in the mean gratitude entries and goal feedback between the 2 trial arms.

##### Sample Size and Effect Size Estimation for Future Definitive Trial

To guide the sample size estimation for a future definitive trial, we used the results of this study. Accordingly, we calculated the expected minimum effect sizes using the primary outcome variable, WEMWBS, and based on the present data, considering the mean change scores for IG and CG and a moderate between-group effect size. Given an *a priori* α of .05, 87 participants were required per group to minimally detect moderate effect sizes (ie, Cohen *f* ≥0.25; Cohen *d* ≥0.5), or 42 participants per group to minimally detect large effect sizes (Cohen f ≥0.4; Cohen *d* ≥0.8), both with a power of 0.95 [36]. Indeed, we must caveat this guide with acknowledgment that the inherent imprecision in between treatment group effect size estimates from studies with small samples can be high and thus must be considered pragmatically in future trials [48].

##### Progression Criteria

All predetermined progression criteria for a definitive trial were met, as described in the sections above.

#### Secondary Outcomes

An ITT analysis was carried out, with missing data rectified by LOCF. The mean scores and SDs for the secondary outcome measures for both trial arms are presented in Table 5. On average, participants in the IG made small improvements from T0 to T1. Participants in the CG showed little to no improvement during the same period. However, after attending the HOPE program (ie, T2), the CG made greater improvements in all secondary measures than the IG.

Table 6 shows the number and proportion of participants exceeding the cutoff scores for probable clinical depression and anxiety at each time point across the trial. In the IG, the number of participants reporting probable clinical levels of depression decreased by 1 from preprogram (8/21, 38%) to postprogram (7/21, 33%), and cases of depression remained the same pre- and postprogram (10/21, 47%). In the CG, at the point of entry into the trial (T0), the proportion of participants reporting probable clinical levels of depression and anxiety was the same (10/20, 50%). From pre- to postprogram in the CG, there was a 15% decrease in cases of probable depression (T1, 7/20, 35%; T2, 4/20, 20%) and anxiety (T1, 8/20, 40%; T2, 5/20, 25%).

**Table 6:** Proportion of participants reporting probable clinical levels of depression and anxiety at each time point across the trial.

Secondary outcome measure	IG^a^, n (%); n=21		CG^b^, n (%); n=20		
	T0	T1^c^	T0	T1^d^	T2^e^
Cases of probable depression	8 (38)	7 (33)	10 (50)	7 (35)	4 (20)
Cases of probable clinical anxiety	10 (47)	10 (47)	10 (50)	8 (40)	5 (25)

^a^IG: intervention group.

^b^CG: control group.

^c^LOCF: last observation carried forward, n=5.

^d^LOCF: last observation carried forward, n=3.

^e^LOCF: last observation carried forward, n=6.

### Ancillary Analyses

We conducted a PP analysis, which included only those participants who completed all study questionnaires and attended at least 3 intervention sessions (PP whole sample n=25; IG n=13, CG n=12). Table 7 shows the secondary outcome measures for the participants. The data in Table 7 show patterns similar to those of the ITT in Table 5. On average, participants in the IG showed modest improvements from T0 to T1. Participants in the CG showed little to no improvement during the same period. The PP analysis shows a difference in the change scores (final column) of greater magnitude compared with the ITT analysis.

**Table 7:** Scores on secondary outcome measures for intervention group (IG; n=13) and control group (CG; n=12) intervention completers (PP), including mean difference in change scores (IG-CG).

Secondary outcome measure	IG (n=13)				CG (n=12)				Difference in change scores Δ IG-CG, mean difference (95% CI)
	T0, mean (SD)	T1, mean (SD)	Postprogram change Δ (T1-T0), mean difference (95% CI)	T0, mean (SD)		T1, mean (SD)	Control change Δ (T1-T0), mean difference (95% CI)		
WEMWBS^a^	44.9 (10.1)	49.5 (12.7)	4.6 (−0.5 to 9.7)	43.8 (12.0)		42.7 (11.7)	−1.1 (−4.7 to 2.5)	5.7 (−0.3 to 11.7)	
PHQ-9^b^	9.1 (6.1)	6.8 (6.5)	−2.2 (−4.3 to −0.2)	9.1 (6.6)		10.8 (6.7)	1.7 (0.4 to 2.9)	−3.9 (−6.2 to −1.6)	
GAD-7^c^	8.6 (6.4)	7.0 (6.6)	−1.6 (−4.4 to 1.2)	8.5 (5.7)		8.8 (4.8)	0.3 (−1.8 to 2.3)	−1.9 (−5.2 to 1.5)	
PAM^d^	63.2 (15.2)	66.2 (14.6)	3.0 (0 to 6.0)	61.6 (12.8)		56.1 (11.2)	−5.5 (−11.7 to 0.7)	8.5 (2.2 to 14.8)	

^a^WEMWBS: Warwick-Edinburgh Mental Well-being Scale.

^b^PHQ-9: 9-item Patient Health Questionnaire.

^c^GAD-7: 7-item Generalized Anxiety Disorder scale.

^d^PAM: Patient Activation Measure.

### Harms

In line with the trial protocol [24], participants who indicated self-harm or suicidal thoughts on the PHQ-9 measure were contacted, along with the MCS administrator, by Hope for the Community (H4C) and were provided with the contact details of local mental health agencies and Samaritans and encouraged to visit their general practitioner. This was the case for 22% (9/41) of participants’ preprogram, and 9% (4/41) of participants postprogram (data not shown in tables). At postprogram, there were no participants who indicated self-harm or suicidal thoughts where they had not already indicated this at the preprogram.

As detailed in the Methods section, participants scoring 10 on the PHQ-9 or 8 on the GAD-7 were categorized as having reached a probable clinical level of depression or anxiety, respectively. Depression was indicated in 44% (18/41) of participants at preprogram and 34% (14/41) at T1. Anxiety was indicated in 49% (20/41) of participants at preprogram and 44% (18/41) at T1. In line with the trial protocol [24], all of these participants were contacted by H4C and encouraged to visit their general practitioner and were signposted to further sources of support as listed above.

At postprogram, there were no participants who reported a probable clinical level of depression where they had not already reported this at the preprogram. However, at postprogram, 5% (2/41) of participants reached a probable clinical level of anxiety but were not previously at this level in the preprogram. Both participants scored 7 on the GAD-7 measure at preprogram, increasing to scores of 8 (n=1) and 9 (n=1) at postprogram. Both participants were contacted by H4C, as outlined above, and in the trial protocol [24]. To provide further context, both participants were in the IG, and only attended 1 (n=1) or 2 (n=1) sessions of the intervention. Both participants were still undergoing treatment for cancer, and one described significant personal stress unrelated to their cancer. Although we cannot rule out the possibility that the intervention may have caused increased anxiety in these 2 participants, they did not engage in more than 2 sessions of the intervention, and the context of the COVID-19 pandemic is linked to increased anxiety among patients with cancer [1,3,5,6]. Furthermore, other participants showed positive changes in their pre- and postprogram mental well-being scores.

## Discussion

### Principal Findings

The feasibility RCT of the digital HOPE program aimed to assess primary outcomes measuring trial feasibility and secondary outcomes relating to measures of participant well-being. The trial yielded encouraging data on the primary outcome measures of recruitment, retention, follow-up, adherence, and engagement rates. More than three-quarters of the participants invited (47/61, 77%) were willing to provide consent and be randomized to either the HOPE program starting the following week, or to a 6-week waiting list. Just over half of the sample (ie, IG and CG combined; 21/41, 51%) completed all 6 sessions of the intervention, and almost two-thirds of the sample (26/41, 63%) completed at least 3 sessions (note that n=1 did not complete the T1 questionnaire, so it was not categorized as an intervention completer. The follow-up rate was encouraging, with a large proportion of participants completing the study questionnaires at T1 (33/41, 80%). Of the participants who completed the T1 questionnaires, 25 also attended 3 intervention sessions, meeting the criteria for intervention completion (25/41, 61%). In terms of engagement, within the sessions, participants viewed between half and three-quarters of the content, on average (range 76%-51%). All of the predetermined progression criteria were met, confirming that a full-scale, fully powered RCT of the digital HOPE program for people with cancer would be feasible.

On average, participants showed increased scores on positive mental well-being and patient activation and decreased scores on anxiety and depression at postprogram relative to baseline. We did not ask participants specific questions relating to their well-being during the COVID-19 pandemic. However, we can tentatively compare data from this study with a previous cohort of people with cancer in the digital HOPE program, collected before the COVID-19 pandemic [23]. The proportion of participants reporting probable clinical levels of depression at baseline was slightly higher in the prepandemic cohort [23] than in the current trial (24/51, 47%, and 18/41, 44%, respectively). However, at baseline, probable clinical levels of anxiety were lower in the prepandemic cohort (22/51, 43%) than in the current trial (20/41, 49%). Indeed, in the current trial, baseline GAD-7 scores for anxiety 8.9 (SD 6.0; pooled data not shown in *Results*) were higher than those in our prepandemic study 6.8 (SD 4.9) [23]. Given the widespread reports of the negative effect of the COVID-19 pandemic on the mental health of people with cancer [3], the baseline scores in the current trial may represent elevated COVID-19–related anxiety.

Exploration of the secondary outcome data highlights the safety of the HOPE program as a digital self-management intervention for people with cancer. There were no cases of participants reporting increased symptoms of depression, and only 2 participants reported increased postprogram anxiety. There were no cases where participants reported thoughts of suicide or harming themselves at postprogram, where they had not already reported this at baseline.

### Limitations

This study found that overall engagement, measured by the percentage of pages viewed, seemed to decline as participants progressed through the sessions. This may be due to fatigue or redundant content. Qualitative investigation into what content participants engaged with, and elements they found more or less relevant or helpful would be a useful supplement to improve the intervention.

This feasibility RCT was not powered to detect statistically significant differences in pre- and postscores on secondary outcomes. However, the results indicate that the HOPE program has the potential to have a positive effect on mental well-being, depression, and anxiety in people with cancer. These have been identified as important outcomes for people with cancer [9-11] and echo the results of a previous pre- and poststudy of the HOPE program [23], giving further confidence in the potential efficacy of the intervention. Data from a fully powered trial will allow us, for the first time, to report statistically significant differences in pre- and postprogram scores for both IG and CG. However, unless we account for expectancy effects, we cannot be sure about the efficacy of the intervention [49]. Therefore, future trials will need to use an appropriate active control program, which equates expectations to those of the IG, to allow a causal conclusion about the effectiveness of the intervention effectiveness. There are a number of ways to address this issue in future work. First, empirical measures of expectancy could be collected before and after the program to assess for any correlation between expectancy and improvement in either group. Although this would not reduce any expectancy effects, it would allow us to account for these in statistical analyses, interpretation of the data, and evaluation of the intervention. Second, in a future trial, we could use an active control program, such as an alternative digital self-management intervention, or a modified version of the HOPE program (eg, without the goal-setting feature; self-directed course). Active control needs to be carefully matched to the intervention in terms of expectancy, content, and interaction [49]. Researchers suggest that if participants’ well-being is improved postintervention, then the mechanisms by which this benefit occurs are irrelevant. However, it becomes a problem if the improvement is only detectable in the laboratory, or in this case, at the time of the postprogram data collection. A third way to address expectancy in future trials would be to implement further follow-up time points (eg, 3, 6, and 12 months postrandomization) to assess whether improvements to participant well-being persist in the longer term. Ideally, in future trials, we would use all 3 methods to reduce placebo effects and increase confidence in the treatment effect of the HOPE program.

### Generalizability

The recruitment for this feasibility RCT was from an opportunity sample of self-selecting participants referred by MCS. The self-selecting nature of the recruitment strategy may yield participants who are generally more motivated to seek help and/or help themselves. However, this recruitment strategy facilitated the rapid attainment of trial recruitment targets in this study [50]. Research has shown that recruitment via social media is more effective if advertised by a collaborative cancer charity [51]. In this respect, in the current climate of increased need for digital research and provision of self-management support, we optimized our recruitment strategy in this feasibility trial and will adopt this again in the definitive trial.

Most participants were White (36/41, 88%), female (32/41, 78.0%), married (30/41, 73%), and educated (24/41, 58%), and the most commonly reported type of cancer was breast cancer (17/41, 41%). This likely relates to the demographics of people who engage with the MCS charity. Although this may limit the generalizability of the results to other demographic groups, some aspects are in line with wider population statistics and research findings. The 2011 Census [52] reported that 86% of the population in England and Wales was White, and therefore, the sample in this study is representative of the wider population in this respect. Breast cancer is the most common type of cancer in the United Kingdom, accounting for 15.1% of malignant cancer registrations in England in 2017 [53]; however, 41.5% of participants in this study reported breast cancer. The data presented in this study may not be representative of other cancer populations. As such, the efficacy signal and feasibility findings of this study may not be generalizable to other types of cancer, or to non-White men, for example. We will seek advice from our partners and trial experts before proceeding to a definitive trial. It may be more appropriate to run a definitive RCT of the HOPE program for breast cancer survivors only, as (1) breast cancer is the most commonly diagnosed cancer in the United Kingdom, and (2) our own data, for example, 23 and unpublished studies, show that it is mainly women with breast cancer who participate in the HOPE program. However, the HOPE program was designed to help people living with all types of cancer; thus, the community HOPE program run by MCS will continue to be open to all cancer survivors.

A low attendance rate for men is common in self-management and is linked to their reluctance to seek help [54]. In terms of recruitment, men are more likely to respond to marketing and recruitment messages that emphasize stoicism, independence, and control [54] and where the materials contain images of men [55]. Once recruited, there are also qualitative differences in how men and women engage with their peers in the same- or mixed-sex web-based cancer support groups [56]. A recent systematic review confirmed that men are more oriented toward informational support and women toward emotional support [57]. In terms of the current intervention, further intervention development is required to ensure the relevance and acceptability of the intervention and potentially to co-design tailored versions for more diverse groups and communities. This may require (1) further consultation with MCS to co-design specific programs for gendered cancers (eg, a HOPE program for testicular, prostrate, or breast cancer); (2) co-development of course content and recruitment materials to increase the engagement of men in a general cancer intervention; and (3) partnering with different charities to enhance engagement with people with cancer from different ethnic groups, socioeconomic status, and educational attainment. A future trial could examine the feasibility of recruitment through the NHS, from clinics, consultation rooms, or waiting rooms, to broaden the recruitment strategy to wider communities. For future cohorts, we will encourage MCS to review their recruitment materials to ensure that they contain images and messages that appeal to multiple audiences and are advertised in (largely web-based) areas and locations frequented by people of all ages, ethnicities, genders, and income groups [54-57].

### Interpretation

The digital HOPE program is a feasible self-management intervention for people with cancer, although almost half of our sample comprised people with breast cancer. All progression criteria were met, providing support for a full-scale definitive trial. However, caution must be taken when interpreting the generalizability of our feasibility estimates, and a further discussion with our research trial group will be undertaken to determine the appropriate action for progression. Generally, at postprogram, all participants showed increased scores on positive mental well-being and patient activation and decreased scores on anxiety and depression relative to baseline, signaling intervention efficacy. Minimal harm was indicated, and no participants reported postprogram symptoms of anxiety or depression that were not present at baseline, and similarly for thoughts of suicide and self-harm.

The asynchronous nature of the course and the autonomy afforded to participants means that they can broadly tailor the course to accommodate personal needs. Participants can attend sessions that are interesting or relevant to them, essentially creating their own person-centered support by self-selecting the content. Rather than interpreting attrition as a negative trial outcome, we concur with the view that participants can be *eAttainers* [35,58]. That is, participants take what they need from the course and then may appear to *drop out* but are nonetheless satisfied and fulfilled by the content they received. Participants do not need to engage with all available components of the intervention, or all use the same components, as goals differ across individuals [35]. For example, some participants obtain reassurance from sharing their challenges and concerns with others, a common group curative factor described as “universality” by Yalom [59]. This approach aligns with the tailored nature of the HOPE program, enabling users to self-select the most relevant intervention tools, features, or content for themselves. This is supported by the overwhelmingly positive user evaluations collected postprogram in this feasibility trial (data not shown) and in a previous HOPE program study [23]. As the focus of this paper was to assess the feasibility of an RCT, and not to evaluate the efficacy and acceptability of the HOPE program [48], we did not present participant feedback data here.

### Conclusions

The advent of the digital HOPE program has coincided with requests from academics, leading cancer charities, and the NHS, for more person-centered cancer self-management, especially during the recent global pandemic [2,3,8]. As such, there has been rapid and essential growth in the provision of health care digitally to allow remote care [60,61], and these advances in digital health care will still be viable in the postpandemic era. This feasibility RCT suggests that the digital HOPE program could supplement in-person emotional and psychological support for people with cancer, offering greater choice and flexibility in accessing support. Although digital cancer self-management interventions, such as the HOPE program, have boomed during COVID-19 [60-62], more data are needed to suggest whether digital could be considered an alternative, as well as complementary, format to in-person intervention programs. As the HOPE program content can be delivered on the web or in-person, it offers flexibility and a choice of formats for participants when social distancing measures are eased after the pandemic.

## Appendix

Multimedia Appendix 1 CONSORT-eHEALTH checklist (V 1.6.1).

## Abbreviations

CG: control group
CONSORT: Consolidated Standards of Reporting Trials
GAD-7: 7-item Generalized Anxiety Disorder scale
H4C: Hope for the Community
HOPE: Help to Overcome Problems Effectively
IG: intervention group
ITT: intention-to-treat
LOCF: last observation carried forward
MCS: Macmillan Cancer Support
NHS: National Health Service
PHQ-9: 9-item Patient Health Questionnaire
PP: per-protocol
RCT: randomized controlled trial
WEMWBS: Warwick Edinburgh Mental Well-being Scale
