# Supplementary material for: A Digital Self-management Program (Help to Overcome Problems Effectively) for People Living With Cancer: Feasibility Randomized Controlled Trial
Source: J Med Internet Res. 2021 Nov 5;23(11):e28322. doi: 10.2196/28322 (PMC8726569; doi:10.2196/28322)
Supplement: Multimedia Appendix 1 [file jmir_v23i11e28322_app1.pdf]

CONSORT-EHEALTH (V 1.6.1) - Submission/Publication Form

The CONSORT-EHEALTH checklist is intended for authors of randomized trials evaluating web-based and Internet-based applications/interventions, including mobile interventions, electronic games (incl multiplayer games), social media, certain telehealth applications, and other interactive and/or networked electronic applications. Some of the items (e.g. all subitems under item 5 - description of the intervention) may also be applicable for other study designs.

The goal of the CONSORT EHEALTH checklist and guideline is to be
a) a guide for reporting for authors of RCTs,
b) to form a basis for appraisal of an ehealth trial (in terms of validity)

CONSORT-EHEALTH items/subitems are MANDATORY reporting items for studies published in the Journal of Medical Internet Research and other journals / scientific societies endorsing the checklist.

Items numbered 1., 2., 3., 4a., 4b etc are original CONSORT or CONSORT-NPT (non-pharmacologic treatment) items.
Items with Roman numerals (i., ii, iii, iv etc.) are CONSORT-EHEALTH extensions/clarifications.

As the CONSORT-EHEALTH checklist is still considered in a formative stage, we would ask that you also RATE ON A SCALE OF 1-5 how important/useful you feel each item is FOR THE PURPOSE OF THE CHECKLIST and reporting guideline (optional).

Mandatory reporting items are marked with a red *.
In the textboxes, either copy & paste the relevant sections from your manuscript into this form - please include any quotes from your manuscript in QUOTATION MARKS,
or answer directly by providing additional information not in the manuscript, or elaborating on why the item was not relevant for this study.

YOUR ANSWERS WILL BE PUBLISHED AS A SUPPLEMENTARY FILE TO YOUR PUBLICATION IN JMIR AND ARE CONSIDERED PART OF YOUR PUBLICATION (IF ACCEPTED).
Please fill in these questions diligently. Information will not be copyedited, so please use proper spelling and grammar, use correct capitalization, and avoid abbreviations.

DO NOT FORGET TO SAVE AS PDF _AND_ CLICK THE SUBMIT BUTTON SO YOUR ANSWERS ARE IN OUR DATABASE !!!

Citation Suggestion (if you append the pdf as Appendix we suggest to cite this paper in the caption):
Eysenbach G, CONSORT-EHEALTH Group
CONSORT-EHEALTH: Improving and Standardizing Evaluation Reports of Web-based and Mobile Health Interventions

J Med Internet Res 2011;13(4):e126
URL: http://www.jmir.org/2011/4/e126/
doi: 10.2196/jmir.1923
PMID: 22209829

Sign in to Google to save your progress. Learn more

* Required

Your name *

First Last

Hayley Wright

Primary Affiliation (short), City, Country *

University of Toronto, Toronto, Canada

Coventry University, Coventry, United Kingdom

Your e-mail address *

abc@gmail.com

ab7764@coventryac.uk

Title of your manuscript *

Provide the (draft) title of your manuscript.

A Digital Self-management Program (Help to Overcome Problems Effectively) for People Living With Cancer: Feasibility Randomized Controlled Trial

Name of your App/Software/Intervention *

If there is a short and a long/alternate name, write the short name first and add the long name in brackets.

The HOPE (Help to Overcome Problems Effect

Evaluated Version (if any)

e.g. "V1", "Release 2017-03-01", "Version 2.0.27913"

Your answer

Language(s) *

What language is the intervention/app in? If multiple languages are available, separate by comma (e.g. "English, French")

English

URL of your Intervention Website or App

e.g. a direct link to the mobile app on app in appstore (itunes, Google Play), or URL of the website. If the intervention is a DVD or hardware, you can also link to an Amazon page.

https://www.h4c.org.uk

URL of an image/screenshot (optional)

Your answer

Accessibility *

Can an enduser access the intervention presently?

- ( ) access is free and open
- (•) access only for special usergroups, not open
- ( ) access is open to everyone, but requires payment/subscription/in-app purchases
- ( ) app/intervention no longer accessible
- ( ) Other:

Primary Medical Indication/Disease/Condition *

e.g. "Stress", "Diabetes", or define the target group in brackets after the condition, e.g. "Autism (Parents of children with)", "Alzheimers (Informal Caregivers of)"

Cancer

Primary Outcomes measured in trial *

comma-separated list of primary outcomes reported in the trial

"Recruitment rates, Retention rates, Follow-up

Secondary/other outcomes

Are there any other outcomes the intervention is expected to affect?

Mental wellbeing, Depression, Anxiety, Patient Activation

Recommended "Dose" *

What do the instructions for users say on how often the app should be used?

- ( ) Approximately Daily
- ( ) Approximately Weekly
- ( ) Approximately Monthly
- ( ) Approximately Yearly
- (x) "as needed"
- ( ) Other:

Approx. Percentage of Users (starters) still using the app as recommended after 3 months *

- ( ) unknown / not evaluated
- ( ) 0-10%
- ( ) 11-20%
- ( ) 21-30%
- ( ) 31-40%
- ( ) 41-50%
- ( ) 51-60%
- ( ) 61-70%
- ( ) 71%-80%
- ( ) 81-90%
- ( ) 91-100%
- (x) Other: 6 week intervention - no longer term follow up was planned for this fea

Overall, was the app/intervention effective? *

- ○ yes: all primary outcomes were significantly better in intervention group vs control
- ○ partly: SOME primary outcomes were significantly better in intervention group vs control
- ○ no statistically significant difference between control and intervention
- ○ potentially harmful: control was significantly better than intervention in one or more outcomes
- ○ inconclusive: more research is needed
- ◉ Other: The study was not powered to detect efficacy, only to test feasibility o

Article Preparation Status/Stage *

At which stage in your article preparation are you currently (at the time you fill in this form)

- ○ not submitted yet - in early draft status
- ○ not submitted yet - in late draft status, just before submission
- ○ submitted to a journal but not reviewed yet
- ○ submitted to a journal and after receiving initial reviewer comments
- ◉ submitted to a journal and accepted, but not published yet
- ○ published
- ○ Other:

Journal *

If you already know where you will submit this paper (or if it is already submitted), please provide the journal name (if it is not JMIR, provide the journal name under "other")

- ○ not submitted yet / unclear where I will submit this
- ◉ Journal of Medical Internet Research (JMIR)
- ○ JMIR mHealth and UHealth
- ○ JMIR Serious Games
- ○ JMIR Mental Health
- ○ JMIR Public Health
- ○ JMIR Formative Research
- ○ Other JMIR sister journal
- ○ Other:

Is this a full powered effectiveness trial or a pilot/feasibility trial? *

- ◉ Pilot/feasibility
- ○ Fully powered

Manuscript tracking number *

If this is a JMIR submission, please provide the manuscript tracking number under "other" (The ms tracking number can be found in the submission acknowledgement email, or when you login as author in JMIR. If the paper is already published in JMIR, then the ms tracking number is the four-digit number at the end of the DOI, to be found at the bottom of each published article in JMIR)

- ○ no ms number (yet) / not (yet) submitted to / published in JMIR
- ◉ Other: #28322

TITLE AND ABSTRACT

1a) TITLE: Identification as a randomized trial in the title

1a) Does your paper address CONSORT item 1a? *

I.e does the title contain the phrase "Randomized Controlled Trial"? (if not, explain the reason under "other")

- (•) yes
- ( ) Other:

1a-i) Identify the mode of delivery in the title

Identify the mode of delivery. Preferably use “web-based” and/or “mobile” and/or “electronic game” in the title. Avoid ambiguous terms like “online”, “virtual”, “interactive”. Use “Internet-based” only if Intervention includes non-web-based Internet components (e.g. email), use “computer-based” or “electronic” only if offline products are used. Use “virtual” only in the context of “virtual reality” (3-D worlds). Use “online” only in the context of “online support groups”. Complement or substitute product names with broader terms for the class of products (such as “mobile” or “smart phone” instead of “iphone”), especially if the application runs on different platforms.

	1	2	3	4	5	
subitem not at all important	(•)	( )	( )	( )	( )	essential

Clear selection

Does your paper address subitem 1a-i? *

Copy and paste relevant sections from manuscript title (include quotes in quotation marks "like this" to indicate direct quotes from your manuscript), or elaborate on this item by providing additional information not in the ms, or briefly explain why the item is not applicable/relevant for your study

We use the term "digital" to describe our program, which can be accessed via any internet-enabled device e.g. smartphone, tablet, laptop

1a-ii) Non-web-based components or important co-interventions in title

Mention non-web-based components or important co-interventions in title, if any (e.g., “with telephone support”).

	1	2	3	4	5	
subitem not at all important	(●)	( )	( )	( )	( )	essential

Clear selection

Does your paper address subitem 1a-ii?

Copy and paste relevant sections from manuscript title (include quotes in quotation marks "like this" to indicate direct quotes from your manuscript), or elaborate on this item by providing additional information not in the ms, or briefly explain why the item is not applicable/relevant for your study

There are no non-web-based components to the program

1a-iii) Primary condition or target group in the title

Mention primary condition or target group in the title, if any (e.g., “for children with Type I Diabetes”) Example: A Web-based and Mobile Intervention with Telephone Support for Children with Type I Diabetes: Randomized Controlled Trial

	1	2	3	4	5	
subitem not at all important	( )	( )	( )	( )	(●)	essential

Clear selection

Does your paper address subitem 1a-iii? *

Copy and paste relevant sections from manuscript title (include quotes in quotation marks "like this" to indicate direct quotes from your manuscript), or elaborate on this item by providing additional information not in the ms, or briefly explain why the item is not applicable/relevant for your study

"A Digital Self-management Program (Help to Overcome Problems Effectively) for People Living With Cancer: Feasibility Randomized Controlled Trial"

1b) ABSTRACT: Structured summary of trial design, methods, results, and conclusions

NPT extension: Description of experimental treatment, comparator, care providers, centers, and blinding status.

1b-i) Key features/functionalities/components of the intervention and comparator in the METHODS section of the ABSTRACT

Mention key features/functionalities/components of the intervention and comparator in the abstract. If possible, also mention theories and principles used for designing the site. Keep in mind the needs of systematic reviewers and indexers by including important synonyms. (Note: Only report in the abstract what the main paper is reporting. If this information is missing from the main body of text, consider adding it)

	1	2	3	4	5	
subitem not at all important	○	◉	○	○	○	essential

Clear selection

Does your paper address subitem 1b-i? *

Copy and paste relevant sections from the manuscript abstract (include quotes in quotation marks "like this" to indicate direct quotes from your manuscript), or elaborate on this item by providing additional information not in the ms, or briefly explain why the item is not applicable/relevant for your study

"Participants were randomised to an intervention group or a waitlist control group. The intervention was a six-week digital self-management program (HOPE Program) for people with cancer"

1b-ii) Level of human involvement in the METHODS section of the ABSTRACT

Clarify the level of human involvement in the abstract, e.g., use phrases like “fully automated” vs. “therapist/nurse/care provider/physician-assisted” (mention number and expertise of providers involved, if any). (Note: Only report in the abstract what the main paper is reporting. If this information is missing from the main body of text, consider adding it)

	1	2	3	4	5	
subitem not at all important	(•)	( )	( )	( )	( )	essential

Clear selection

Does your paper address subitem 1b-ii?

Copy and paste relevant sections from the manuscript abstract (include quotes in quotation marks "like this" to indicate direct quotes from your manuscript), or elaborate on this item by providing additional information not in the ms, or briefly explain why the item is not applicable/relevant for your study

Participants were supported by a peer facilitator throughout the program. Peer facilitators respond to queries, stimulate conversations and encourage participants to support each other, via the in-program discussion forums, private messaging, and feedback functions.

1b-iii) Open vs. closed, web-based (self-assessment) vs. face-to-face assessments in the METHODS section of the ABSTRACT

Mention how participants were recruited (online vs. offline), e.g., from an open access website or from a clinic or a closed online user group (closed usergroup trial), and clarify if this was a purely web-based trial, or there were face-to-face components (as part of the intervention or for assessment). Clearly say if outcomes were self-assessed through questionnaires (as common in web-based trials). Note: In traditional offline trials, an open trial (open-label trial) is a type of clinical trial in which both the researchers and participants know which treatment is being administered. To avoid confusion, use “blinded” or “unblinded” to indicated the level of blinding instead of “open”, as “open” in web-based trials usually refers to “open access” (i.e. participants can self-enrol). (Note: Only report in the abstract what the main paper is reporting. If this information is missing from the main body of text, consider adding it)

	1	2	3	4	5	
subitem not at all important	( )	(•)	( )	( )	( )	essential

Clear selection

Does your paper address subitem 1b-iii?

Copy and paste relevant sections from the manuscript abstract (include quotes in quotation marks "like this" to indicate direct quotes from your manuscript), or elaborate on this item by providing additional information not in the ms, or briefly explain why the item is not applicable/relevant for your study

"Participants were drawn from an opportunity sample, referred by Macmillan Cancer Support and were invited via email to take part in the study"

1b-iv) RESULTS section in abstract must contain use data

Report number of participants enrolled/assessed in each group, the use/uptake of the intervention (e.g., attrition/adherence metrics, use over time, number of logins etc.), in addition to primary/secondary outcomes. (Note: Only report in the abstract what the main paper is reporting. If this information is missing from the main body of text, consider adding it)

	1	2	3	4	5	
subitem not at all important	○	○	○	○	◉	essential

Clear selection

Does your paper address subitem 1b-iv?

Copy and paste relevant sections from the manuscript abstract (include quotes in quotation marks "like this" to indicate direct quotes from your manuscript), or elaborate on this item by providing additional information not in the ms, or briefly explain why the item is not applicable/relevant for your study

"The recruitment rate was 77% (N=47). Forty-one participants (n=41) completed the baseline questionnaires and were randomised to either the intervention group (n=21) or waitlist control group (n=20). The retention rate (attending all program sessions) was over 50% (all n=21, 51.2%; intervention group n=10, 47.6%; control group n=11, 55.0%), the follow up rate (completing all questionnaires) was over 80% (all 80.5%, n=33; intervention group 76.2%, n=16; control group 85.0%, n=17), and completion rate (attending [illegible]3 sessions and completing all questionnaires) was over 60% (all n=25, 61.0%; intervention group n=13, 61.9%; control group n=12, 60.0%). Engagement data showed that participants viewed between half (n=5.1, 51.0%) and three quarters (n=12.2, 76.3%) of the pages in each session"

1b-v) CONCLUSIONS/DISCUSSION in abstract for negative trials

Conclusions/Discussions in abstract for negative trials: Discuss the primary outcome - if the trial is negative (primary outcome not changed), and the intervention was not used, discuss whether negative results are attributable to lack of uptake and discuss reasons. (Note: Only report in the abstract what the main paper is reporting. If this information is missing from the main body of text, consider adding it)

	1	2	3	4	5	
subitem not at all important	(●)	( )	( )	( )	( )	essential

Clear selection

Does your paper address subitem 1b-v?

Copy and paste relevant sections from the manuscript abstract (include quotes in quotation marks "like this" to indicate direct quotes from your manuscript), or elaborate on this item by providing additional information not in the ms, or briefly explain why the item is not applicable/relevant for your study

"All progression criteria for a definitive trial were met, as supported by the primary outcome data. The intervention group showed improved postprogram scores on measures of positive mental wellbeing, depression, anxiety and patient activation. A full-scale trial of the digital HOPE Program for people with cancer will allow us to fully evaluate the efficacy of the intervention relative to a control group"

INTRODUCTION

2a) In INTRODUCTION: Scientific background and explanation of rationale

2a-i) Problem and the type of system/solution

Describe the problem and the type of system/solution that is object of the study: intended as stand-alone intervention vs. incorporated in broader health care program? Intended for a particular patient population? Goals of the intervention, e.g., being more cost-effective to other interventions, replace or complement other solutions? (Note: Details about the intervention are provided in “Methods” under 5)

	1	2	3	4	5	
subitem not at all important	○	○	○	◉	○	essential

Clear selection

Does your paper address subitem 2a-i? *

Copy and paste relevant sections from the manuscript (include quotes in quotation marks "like this" to indicate direct quotes from your manuscript), or elaborate on this item by providing additional information not in the ms, or briefly explain why the item is not applicable/relevant for your study

"Initial evaluation has suggested positive effects on anxiety, depression and positive wellbeing in people with cancer, with positive user feedback [23]. This suggested that a trial of the digital HOPE Program might be viable and meaningful. A feasibility randomised controlled trial (RCT) study of the digital intervention was required to assess whether participants consent to being randomised, and to test the feasibility of running a wait-list control study design of the HOPE Program"

2a-ii) Scientific background, rationale: What is known about the (type of) system

Scientific background, rationale: What is known about the (type of) system that is the object of the study (be sure to discuss the use of similar systems for other conditions/diagnoses, if appropiate), motivation for the study, i.e. what are the reasons for and what is the context for this specific study, from which stakeholder viewpoint is the study performed, potential impact of findings [2]. Briefly justify the choice of the comparator.

	1	2	3	4	5	
subitem not at all important	○	○	◉	○	○	essential

Clear selection

Does your paper address subitem 2a-ii? *

Copy and paste relevant sections from the manuscript (include quotes in quotation marks "like this" to indicate direct quotes from your manuscript), or elaborate on this item by providing additional information not in the ms, or briefly explain why the item is not applicable/relevant for your study

"Before the COVID-19 pandemic, there was a shortage of accessible self-management interventions, and there is even greater need for digital interventions now to comply with social distancing guidelines"

2b) In INTRODUCTION: Specific objectives or hypotheses

Does your paper address CONSORT subitem 2b? *

Copy and paste relevant sections from the manuscript (include quotes in quotation marks "like this" to indicate direct quotes from your manuscript), or elaborate on this item by providing additional information not in the ms, or briefly explain why the item is not applicable/relevant for your study

The specific objectives were related to the feasibility of an RCT

METHODS

3a) Description of trial design (such as parallel, factorial) including allocation ratio

Does your paper address CONSORT subitem 3a? *

Copy and paste relevant sections from the manuscript (include quotes in quotation marks "like this" to indicate direct quotes from your manuscript), or elaborate on this item by providing additional information not in the ms, or briefly explain why the item is not applicable/relevant for your study

"This study employed a feasibility, randomised waitlist control group parallel design, with a 1:1 allocation ratio. Participants were randomised to an intervention group (IG) or a waitlist control group (CG). The IG received access to the digital six-week HOPE Program immediately. The CG were placed on a waiting list for approximately six weeks, after which time they also received access to the same digital six-week HOPE Program. Key outcome measures were collected via online questionnaires at Time 0 (T0; baseline) and Time 1 (T1; 6 weeks post-randomisation and postprogram for IG). We also sent the questionnaires to the CG only again after they had received the intervention (Time 2; T2; postprogram for CG)"

3b) Important changes to methods after trial commencement (such as eligibility criteria), with reasons

Does your paper address CONSORT subitem 3b? *

Copy and paste relevant sections from the manuscript (include quotes in quotation marks "like this" to indicate direct quotes from your manuscript), or elaborate on this item by providing additional information not in the ms, or briefly explain why the item is not applicable/relevant for your study

No changes were made to the methods after trial commencement

3b-i) Bug fixes, Downtimes, Content Changes

Bug fixes, Downtimes, Content Changes: ehealth systems are often dynamic systems. A description of changes to methods therefore also includes important changes made on the intervention or comparator during the trial (e.g., major bug fixes or changes in the functionality or content) (5-iii) and other “unexpected events” that may have influenced study design such as staff changes, system failures/downtimes, etc. [2].

	1	2	3	4	5	
subitem not at all important	○	○	○	◉	○	essential

Clear selection

Does your paper address subitem 3b-i?

Copy and paste relevant sections from the manuscript (include quotes in quotation marks "like this" to indicate direct quotes from your manuscript), or elaborate on this item by providing additional information not in the ms, or briefly explain why the item is not applicable/relevant for your study

The study was not affected by any of the above issues

4a) Eligibility criteria for participants

Does your paper address CONSORT subitem 4a? *

Copy and paste relevant sections from the manuscript (include quotes in quotation marks "like this" to indicate direct quotes from your manuscript), or elaborate on this item by providing additional information not in the ms, or briefly explain why the item is not applicable/relevant for your study

"Any cancer diagnosis, at any treatment stage, Adult (18 years or over), Located in the United Kingdom, Access to the internet and a device that allows them to engage with the intervention, Fluent in English to be able to engage with all the material in the intervention"

4a-i) Computer / Internet literacy

Computer / Internet literacy is often an implicit "de facto" eligibility criterion - this should be explicitly clarified.

	1	2	3	4	5	
subitem not at all important	○	○	○	◉	○	essential

Clear selection

Does your paper address subitem 4a-i?

Copy and paste relevant sections from the manuscript (include quotes in quotation marks "like this" to indicate direct quotes from your manuscript), or elaborate on this item by providing additional information not in the ms, or briefly explain why the item is not applicable/relevant for your study

"Access to the internet and a device that allows them to engage with the intervention, Fluent in English to be able to engage with all the material in the intervention"

4a-ii) Open vs. closed, web-based vs. face-to-face assessments:

Open vs. closed, web-based vs. face-to-face assessments: Mention how participants were recruited (online vs. offline), e.g., from an open access website or from a clinic, and clarify if this was a purely web-based trial, or there were face-to-face components (as part of the intervention or for assessment), i.e., to what degree got the study team to know the participant. In online-only trials, clarify if participants were quasi-anonymous and whether having multiple identities was possible or whether technical or logistical measures (e.g., cookies, email confirmation, phone calls) were used to detect/prevent these.

	1	2	3	4	5	
subitem not at all important	○	○	○	●	○	essential

Clear selection

Does your paper address subitem 4a-ii? *

Copy and paste relevant sections from the manuscript (include quotes in quotation marks "like this" to indicate direct quotes from your manuscript), or elaborate on this item by providing additional information not in the ms, or briefly explain why the item is not applicable/relevant for your study

"Participants were referred by Macmillan Cancer Support (MCS), a leading UK cancer charity. They advertise the HOPE Program through their social media networks, MCS websites, and word of mouth through specialist nurses"

4a-iii) Information giving during recruitment

Information given during recruitment. Specify how participants were briefed for recruitment and in the informed consent procedures (e.g., publish the informed consent documentation as appendix, see also item X26), as this information may have an effect on user self-selection, user expectation and may also bias results.

	1	2	3	4	5	
subitem not at all important	○	○	○	○	●	essential

Clear selection

Does your paper address subitem 4a-iii?

Copy and paste relevant sections from the manuscript (include quotes in quotation marks "like this" to indicate direct quotes from your manuscript), or elaborate on this item by providing additional information not in the ms, or briefly explain why the item is not applicable/relevant for your study

The informed consent form and participant information sheet are published in the Protocol for this trial (see Multimedia Appendix 2), which can be accessed here: https://www.researchprotocols.org/2020/12/e24264

4b) Settings and locations where the data were collected

Does your paper address CONSORT subitem 4b? *

Copy and paste relevant sections from the manuscript (include quotes in quotation marks "like this" to indicate direct quotes from your manuscript), or elaborate on this item by providing additional information not in the ms, or briefly explain why the item is not applicable/relevant for your study

"The intervention and data collection took place online"

4b-i) Report if outcomes were (self-)assessed through online questionnaires

Clearly report if outcomes were (self-)assessed through online questionnaires (as common in web-based trials) or otherwise.

	1	2	3	4	5	
subitem not at all important	○	○	○	◉	○	essential

Clear selection

Does your paper address subitem 4b-i? *

Copy and paste relevant sections from the manuscript (include quotes in quotation marks "like this" to indicate direct quotes from your manuscript), or elaborate on this item by providing additional information not in the ms, or briefly explain why the item is not applicable/relevant for your study

"All study data were collected online via questionnaires administered through Qualtrics Survey Software (Qualtrics 2019, Provo, UT, USA, Available from: http://www.qualtrics.com)"

4b-ii) Report how institutional affiliations are displayed

Report how institutional affiliations are displayed to potential participants [on ehealth media], as affiliations with prestigious hospitals or universities may affect volunteer rates, use, and reactions with regards to an intervention.(Not a required item – describe only if this may bias results)

	1	2	3	4	5	
subitem not at all important	○	○	◉	○	○	essential

Clear selection

Does your paper address subitem 4b-ii?

Copy and paste relevant sections from the manuscript (include quotes in quotation marks "like this" to indicate direct quotes from your manuscript), or elaborate on this item by providing additional information not in the ms, or briefly explain why the item is not applicable/relevant for your study

The Coventry University and Macmillan Cancer Support logos were displayed on a study banner across the online participant survey.

5) The interventions for each group with sufficient details to allow replication, including how and when they were actually administered

5-i) Mention names, credential, affiliations of the developers, sponsors, and owners

Mention names, credential, affiliations of the developers, sponsors, and owners [6] (if authors/evaluators are owners or developer of the software, this needs to be declared in a "Conflict of interest" section or mentioned elsewhere in the manuscript).

	1	2	3	4	5	
subitem not at all important	○	○	◉	○	○	essential

Clear selection

Does your paper address subitem 5-i?

Copy and paste relevant sections from the manuscript (include quotes in quotation marks "like this" to indicate direct quotes from your manuscript), or elaborate on this item by providing additional information not in the ms, or briefly explain why the item is not applicable/relevant for your study

The digital platform for the HOPE Program is owned by Hope For The Community (H4C) Community Interest Company, a small UK-based social enterprise. H4C is a spin-out company of Coventry University. Co-author Andy Turner is co-founder of the HOPE Program and non-executive director of H4C. Co-author Gabriela Matouskova is CEO of H4C.

5-ii) Describe the history/development process

Describe the history/development process of the application and previous formative evaluations (e.g., focus groups, usability testing), as these will have an impact on adoption/use rates and help with interpreting results.

	1	2	3	4	5	
subitem not at all important	○	○	○	◉	○	essential

Clear selection

Does your paper address subitem 5-ii?

Copy and paste relevant sections from the manuscript (include quotes in quotation marks "like this" to indicate direct quotes from your manuscript), or elaborate on this item by providing additional information not in the ms, or briefly explain why the item is not applicable/relevant for your study

"Around a decade ago, we co-designed a face-to-face self-management program for survivors of all types of cancer [21,22]. People with cancer, oncologists, specialist cancer nurses, and representatives from a leading UK cancer charity (Macmillan Cancer Support; MCS) were involved in the co-design process. This led to the “Help to Overcome Problems Effectively” (HOPE) Program, which has been described in detail elsewhere [23,24]. The HOPE program aims to enhance well-being by fostering positive emotions and stimulate positive functioning. A parallel goal is to reduce depressive symptoms. The HOPE program is based on principles derived from positive psychology and focuses on positive experiences, strengths, and personal competencies rather than mental health problems such as anxiety and depression. It incorporates evidence-based exercises based on positive psychology, in addition to elements stemming from mindfulness, cognitive behavioural therapy and problem-solving therapy. The HOPE Program recognises the common challenges and unmet needs across all types of cancer including fatigue, fear of recurrence and psychological distress [9-20]. The Hope Program was designed to provide support for these most common, typically overlapping needs, in people living with most types of cancer. We regularly consult with MCS on their eHealth Needs Assessment data, and review the most common needs indicated by people living with all types of cancer. The Hope Program provides general psychological and wellbeing support based on these needs, and is open to all adult cancer survivors. The HOPE Program differs from many other cancer self-management programs due to the focus on i) positive psychology [25-27], and ii) hope and gratitude [28] to improve wellbeing and coping, the iii) co-created content, and iv) peer facilitated delivery. The HOPE Program is moderated by trained peer facilitators who themselves are affected by cancer in some way. The facilitators have received training from Macmillan Cancer Support and follow a delivery protocol. The facilitator’s role is to offer encouragement to participants, stimulate discussion in social networking forums by inviting participants to respond with comments to specific questions, or respond to questions or comments posted by participants. Facilitators also monitor the daily social networking posts for safety and report any technical problems to the research team. Facilitators spent approximately two hours per session supporting the participants. The in-person program was adapted for digital delivery (see [24] for full details of adaptation), employing a user-centred, iterative approach [29]. A set of design requirements and a design brief were drawn up in consultation with end-users and stakeholders. The initial digital version of HOPE went through a number of iterative testing sessions, with improvements made to usability after each iteration. It was intended through these iterations to develop a system that was useable and accepted by the intended user group to increase the likelihood of uptake and continued usage, and ensure the technology did not prove a barrier to engagement and participation"

5-iii) Revisions and updating

Revisions and updating. Clearly mention the date and/or version number of the application/intervention (and comparator, if applicable) evaluated, or describe whether the intervention underwent major changes during the evaluation process, or whether the development and/or content was “frozen” during the trial. Describe dynamic components such as news feeds or changing content which may have an impact on the replicability of the intervention (for unexpected events see item 3b).

	1	2	3	4	5	
subitem not at all important	○	○	○	◉	○	essential

Clear selection

Does your paper address subitem 5-iii?

Copy and paste relevant sections from the manuscript (include quotes in quotation marks "like this" to indicate direct quotes from your manuscript), or elaborate on this item by providing additional information not in the ms, or briefly explain why the item is not applicable/relevant for your study

The content was frozen during the trial.

5-iv) Quality assurance methods

Provide information on quality assurance methods to ensure accuracy and quality of information provided [1], if applicable.

	1	2	3	4	5	
subitem not at all important	○	○	○	◉	○	essential

Clear selection

Does your paper address subitem 5-iv?

Copy and paste relevant sections from the manuscript (include quotes in quotation marks "like this" to indicate direct quotes from your manuscript), or elaborate on this item by providing additional information not in the ms, or briefly explain why the item is not applicable/relevant for your study

We address intervention fidelity through training and monitoring of facilitators, and providing a manual for reference "The facilitators have received training from Macmillan Cancer Support and follow a delivery protocol ... Facilitators also monitor the daily social networking posts for safety and report any technical problems to the research team"
The HOPE Program content "incorporates evidence-based exercises based on positive psychology, in addition to elements stemming from mindfulness, cognitive behavioural therapy and problem-solving therapy...We regularly consult with MCS on their eHealth Needs Assessment data, and review the most common needs indicated by people living with all types of cancer. The Hope Program provides general psychological and wellbeing support based on these needs, and is open to all adult cancer survivors"

5-v) Ensure replicability by publishing the source code, and/or providing screenshots/screen-capture video, and/or providing flowcharts of the algorithms used

Ensure replicability by publishing the source code, and/or providing screenshots/screen-capture video, and/or providing flowcharts of the algorithms used. Replicability (i.e., other researchers should in principle be able to replicate the study) is a hallmark of scientific reporting.

	1	2	3	4	5	
subitem not at all important	○	○	○	●	○	essential

Clear selection

Does your paper address subitem 5-v?

Copy and paste relevant sections from the manuscript (include quotes in quotation marks "like this" to indicate direct quotes from your manuscript), or elaborate on this item by providing additional information not in the ms, or briefly explain why the item is not applicable/relevant for your study

We provide screenshots of the HOPE Program in our previous paper published in JMIR which can be accessed here: https://www.jmir.org/2020/5/e17824/

5-vi) Digital preservation

Digital preservation: Provide the URL of the application, but as the intervention is likely to change or disappear over the course of the years; also make sure the intervention is archived (Internet Archive, webcitation.org, and/or publishing the source code or screenshots/videos alongside the article). As pages behind login screens cannot be archived, consider creating demo pages which are accessible without login.

	1	2	3	4	5	
subitem not at all important	○	◉	○	○	○	essential

Clear selection

Does your paper address subitem 5-vi?

Copy and paste relevant sections from the manuscript (include quotes in quotation marks "like this" to indicate direct quotes from your manuscript), or elaborate on this item by providing additional information not in the ms, or briefly explain why the item is not applicable/relevant for your study

"All the HOPE Program modules have the same structure and format, with a variety of components each week focussing on a particular issue or set of techniques, and ending with a goal setting activity. The HOPE Program is asynchronous, and content is released on a weekly basis at set times (e.g. at midday every Monday) over the six weeks of the intervention"
H4C can provide demo pages for the HOPE Program on request. Please email hope@h4c.org.uk

5-vii) Access

Access: Describe how participants accessed the application, in what setting/context, if they had to pay (or were paid) or not, whether they had to be a member of specific group. If known, describe how participants obtained “access to the platform and Internet” [1]. To ensure access for editors/reviewers/readers, consider to provide a “backdoor” login account or demo mode for reviewers/readers to explore the application (also important for archiving purposes, see vi).

	1	2	3	4	5	
subitem not at all important	○	○	○	○	◉	essential

Clear selection

Does your paper address subitem 5-vii? *

Copy and paste relevant sections from the manuscript (include quotes in quotation marks "like this" to indicate direct quotes from your manuscript), or elaborate on this item by providing additional information not in the ms, or briefly explain why the item is not applicable/relevant for your study

"Participants were referred [to the HOPE Program] by Macmillan Cancer Support (MCS), a leading UK cancer charity. They advertise the HOPE Program through their social media networks, MCS websites, and word of mouth through specialist nurses...Participants were randomised to an intervention group (IG) or a waitlist control group (CG). The IG received access to the digital six-week HOPE Program immediately. The CG were placed on a waiting list for approximately six weeks, after which time they also received access to the same digital six-week HOPE Program"
Participants were not paid for taking part, but were entered into a prize draw for a voucher if they completed all study questionnaires.

5-viii) Mode of delivery, features/functionalities/components of the intervention and comparator, and the theoretical framework

Describe mode of delivery, features/functionalities/components of the intervention and comparator, and the theoretical framework [6] used to design them (instructional strategy [1], behaviour change techniques, persuasive features, etc., see e.g., [7, 8] for terminology). This includes an in-depth description of the content (including where it is coming from and who developed it) [1],” whether [and how] it is tailored to individual circumstances and allows users to track their progress and receive feedback” [6]. This also includes a description of communication delivery channels and – if computer-mediated communication is a component – whether communication was synchronous or asynchronous [6]. It also includes information on presentation strategies [1], including page design principles, average amount of text on pages, presence of hyperlinks to other resources, etc. [1].

	1	2	3	4	5	
subitem not at all important	◉	○	○	○	○	essential

Clear selection

Does your paper address subitem 5-viii? *

Copy and paste relevant sections from the manuscript (include quotes in quotation marks "like this" to indicate direct quotes from your manuscript), or elaborate on this item by providing additional information not in the ms, or briefly explain why the item is not applicable/relevant for your study

"The HOPE Program was delivered online. Full details of the digital HOPE Program development, content and weekly topics have been described elsewhere (see [23,24]) but we provide a brief overview here. All the HOPE Program modules have the same structure and format, with a variety of components each week focussing on a particular issue or set of techniques, and ending with a goal setting activity. The HOPE Program is asynchronous, and content is released on a weekly basis at set times (e.g. at midday every Monday) over the six weeks of the intervention. Forums and messaging facilities acted as a conduit for communication between participants and facilitators, and the Program was moderated by trained peer facilitators. Table 1 gives an overview of the content and activities within each weekly module of the HOPE Program"

5-ix) Describe use parameters

Describe use parameters (e.g., intended “doses” and optimal timing for use). Clarify what instructions or recommendations were given to the user, e.g., regarding timing, frequency, heaviness of use, if any, or was the intervention used ad libitum.

	1	2	3	4	5	
subitem not at all important	○	○	○	◉	○	essential

Clear selection

Does your paper address subitem 5-ix?

Copy and paste relevant sections from the manuscript (include quotes in quotation marks "like this" to indicate direct quotes from your manuscript), or elaborate on this item by providing additional information not in the ms, or briefly explain why the item is not applicable/relevant for your study

"The intervention platform collects user engagement data such as number of pages viewed in each session and number of goals set which assists the moderators with participant engagement and experience. We measured the mean percentage of pages viewed per session, and number of posts/comments a participant made for key activities (gratitude, setting goals, goal feedback, liking posts, and comments posted)"
"Participant retention rate was calculated by the percentage of participants attending all six program sessions. Studies show that a median of 56% of participants complete the full programme in digital interventions for mental wellbeing [31,32]. As high rates of non-usage attrition [33] are common and of concern in digitally delivered interventions, and because of uncertainties relating to the COVID-19 pandemic, we set a more conservative target of 50% of participants completing all 6 sessions of the intervention... If participants attended at least half of the intervention ([illegible]3 sessions) [31] and completed the study questionnaires, they were classed as intervention completers. Studies show a non-linear relationship between time spent on an intervention, the number of sessions completed, and outcomes [31]. Amount of usage needed to obtain desired outcomes varies across groups, and individuals may stop using the intervention once personal goals are achieved [34]. Therefore, we set a more pragmatic target for our primary outcome measure of 'completion rate', of at least 3 sessions attended and all study questionnaires completed"

5-x) Clarify the level of human involvement

Clarify the level of human involvement (care providers or health professionals, also technical assistance) in the e-intervention or as co-intervention (detail number and expertise of professionals involved, if any, as well as "type of assistance offered, the timing and frequency of the support, how it is initiated, and the medium by which the assistance is delivered". It may be necessary to distinguish between the level of human involvement required for the trial, and the level of human involvement required for a routine application outside of a RCT setting (discuss under item 21 – generalizability).

	1	2	3	4	5	
subitem not at all important	○	○	●	○	○	essential

Clear selection

Does your paper address subitem 5-x?

Copy and paste relevant sections from the manuscript (include quotes in quotation marks "like this" to indicate direct quotes from your manuscript), or elaborate on this item by providing additional information not in the ms, or briefly explain why the item is not applicable/relevant for your study

"The HOPE Program is moderated by trained peer facilitators who themselves are affected by cancer in some way. The facilitators have received training from Macmillan Cancer Support and follow a delivery protocol. The facilitator’s role is to offer encouragement to participants, stimulate discussion in social networking forums by inviting participants to respond with comments to specific questions, or respond to questions or comments posted by participants. Facilitators also monitor the daily social networking posts for safety and report any technical problems to the research team. Facilitators spent approximately two hours per session supporting the participants"

5-xi) Report any prompts/reminders used

Report any prompts/reminders used: Clarify if there were prompts (letters, emails, phone calls, SMS) to use the application, what triggered them, frequency etc. It may be necessary to distinguish between the level of prompts/reminders required for the trial, and the level of prompts/reminders for a routine application outside of a RCT setting (discuss under item 21 – generalizability).

	1	2	3	4	5	
subitem not at all important	○	○	◉	○	○	essential

Clear selection

Does your paper address subitem 5-xi? *

Copy and paste relevant sections from the manuscript (include quotes in quotation marks "like this" to indicate direct quotes from your manuscript), or elaborate on this item by providing additional information not in the ms, or briefly explain why the item is not applicable/relevant for your study

Participants receive an email each week to inform them that new Program content is available. Automated email prompts are sent if participants have not logged in to the intervention for a period of time e.g. one week.

Participants are emailed a link to the study questionnaires (at T0, T1, T2), and two automated reminders are sent via email if participants have not accessed the questionnaires e.g. after 7 days and after 14 days.

5-xii) Describe any co-interventions (incl. training/support)

Describe any co-interventions (incl. training/support): Clearly state any interventions that are provided in addition to the targeted eHealth intervention, as ehealth intervention may not be designed as stand-alone intervention. This includes training sessions and support [1]. It may be necessary to distinguish between the level of training required for the trial, and the level of training for a routine application outside of a RCT setting (discuss under item 21 – generalizability.

	1	2	3	4	5	
subitem not at all important	○	◉	○	○	○	essential

Clear selection

Does your paper address subitem 5-xii? *

Copy and paste relevant sections from the manuscript (include quotes in quotation marks "like this" to indicate direct quotes from your manuscript), or elaborate on this item by providing additional information not in the ms, or briefly explain why the item is not applicable/relevant for your study

Peer facilitators receive formal training and assessment (QISMET) in self management support and health coaching. Peer facilitators spend approximately 2 hours per week supporting participants on the online course.

6a) Completely defined pre-specified primary and secondary outcome measures, including how and when they were assessed

Does your paper address CONSORT subitem 6a? *

Copy and paste relevant sections from the manuscript (include quotes in quotation marks "like this" to indicate direct quotes from your manuscript), or elaborate on this item by providing additional information not in the ms, or briefly explain why the item is not applicable/relevant for your study

"The planned primary outcomes (trail feasibility objectives) of the study were to establish the following:

- Recruitment rates for participation and for randomization
- Retention and follow-up rates as the participants move through the trial
- Adherence rates to study procedures, intervention attendance, and engagement
- Sample size and effect size estimation for a definitive trial
- Progression criteria for a definitive trial"

Primary outcomes were assessed by inspection of the data after the 6 week intervention.

"Recruitment rates were then calculated from those a) providing consent, b) completing baseline questionnaires. A direct email from participants indicating refusal or declining to participate in the study, indicated a refusal."

"Participant retention rate was calculated by the percentage of participants attending all six program sessions"

"Follow up was calculated by the percentage of participants who completed all the online study questionnaires"

"If participants attended at least half of the intervention ([illegible]3 sessions) [31] and completed the study questionnaires, they were classed as intervention completers"

"The secondary outcomes related to participant wellbeing:

- Measures of positive mental well-being [Warwick Edinburgh Mental Wellbeing Scale (WEMWBS)], depression [Patient Health Questionnaire (PHQ-9)], anxiety [Generalized Anxiety Disorder scale (GAD 7)], and confidence to self-manage cancer (patient activation) [Patient Activation Measure (PAM®)], as indicated by scores on validated measures"

Secondary outcome measures were assessed via validated questionnaires at baseline (T0) and 6-weeks post-randomisation. The control group completed them again at the end of the 6 week program. All questionnaires were administered online via Qualtrics.

6a-i) Online questionnaires: describe if they were validated for online use and apply CHERRIES items to describe how the questionnaires were designed/deployed

If outcomes were obtained through online questionnaires, describe if they were validated for online use and apply CHERRIES items to describe how the questionnaires were designed/deployed [9].

	1	2	3	4	5	
subitem not at all important	◉	○	○	○	○	essential

Clear selection

Does your paper address subitem 6a-i?

Copy and paste relevant sections from manuscript text

Your answer

6a-ii) Describe whether and how “use” (including intensity of use/dosage) was defined/measured/monitored

Describe whether and how “use” (including intensity of use/dosage) was defined/measured/monitored (logins, logfile analysis, etc.). Use/adoption metrics are important process outcomes that should be reported in any ehealth trial.

	1	2	3	4	5	
subitem not at all important	○	○	○	◉	○	essential

Clear selection

Does your paper address subitem 6a-ii?

Copy and paste relevant sections from manuscript text

"Participant retention rate was calculated by the percentage of participants attending all six program sessions. Studies show that a median of 56% of participants complete the full programme in digital interventions for mental wellbeing [31,32]. As high rates of non-usage attrition [33] are common and of concern in digitally delivered interventions, and because of uncertainties relating to the COVID-19 pandemic, we set a more conservative target of 50% of participants completing all 6 sessions of the intervention...If participants attended at least half of the intervention ([illegible]3 sessions) [31] and completed the study questionnaires, they were classed as intervention completers. Studies show a non-linear relationship between time spent on an intervention, the number of sessions completed, and outcomes [31]. Amount of usage needed to obtain desired outcomes varies across groups, and individuals may stop using the intervention once personal goals are achieved [34]. Therefore, we set a more pragmatic target for our primary outcome measure of 'completion rate', of at least 3 sessions attended and all study questionnaires completed"

"The intervention platform collects user engagement data such as number of pages viewed in each session and number of goals set which assists the moderators with participant engagement and experience. We measured the mean percentage of pages viewed per session, and number of posts/comments a participant made for key activities (gratitude, setting goals, goal feedback, liking posts, and comments posted)"

6a-iii) Describe whether, how, and when qualitative feedback from participants was obtained

Describe whether, how, and when qualitative feedback from participants was obtained (e.g., through emails, feedback forms, interviews, focus groups).

	1	2	3	4	5	
subitem not at all important	○	○	○	●	○	essential

Clear selection

Does your paper address subitem 6a-iii?

Copy and paste relevant sections from manuscript text

Participants could provide free-text responses on the usability feedback questionnaire post-program. However, we do not present feedback data in this paper.

6b) Any changes to trial outcomes after the trial commenced, with reasons

Does your paper address CONSORT subitem 6b? *

Copy and paste relevant sections from the manuscript (include quotes in quotation marks "like this" to indicate direct quotes from your manuscript), or elaborate on this item by providing additional information not in the ms, or briefly explain why the item is not applicable/relevant for your study

No changes were made to trial outcomes after the trial commenced.

7a) How sample size was determined

NPT: When applicable, details of whether and how the clustering by care provides or centers was addressed

7a-i) Describe whether and how expected attrition was taken into account when calculating the sample size

Describe whether and how expected attrition was taken into account when calculating the sample size.

	1	2	3	4	5	
subitem not at all important	○	○	○	○	●	essential

Clear selection

Does your paper address subitem 7a-i?

Copy and paste relevant sections from manuscript title (include quotes in quotation marks "like this" to indicate direct quotes from your manuscript), or elaborate on this item by providing additional information not in the ms, or briefly explain why the item is not applicable/relevant for your study

"All study participants were drawn from an opportunity sample (N=61), provided by MCS, of eligible candidates who had expressed an interest in taking part in the HOPE Program. An arbitrary sample size of n=40 was deemed adequate for this feasibility study, informed by similar studies in this area with sample sizes ranging from n=10 to n=20 in each arm [44]. All potential study participants were emailed a link to the study website hosted by Qualtrics, where they were asked to read the digital Participant Information Sheet (PIS), read and agree to the statements on the digital consent form, and complete the digital T0 questionnaire, before randomisation"

7b) When applicable, explanation of any interim analyses and stopping guidelines

Does your paper address CONSORT subitem 7b? *

Copy and paste relevant sections from the manuscript (include quotes in quotation marks "like this" to indicate direct quotes from your manuscript), or elaborate on this item by providing additional information not in the ms, or briefly explain why the item is not applicable/relevant for your study

Not applicable as this was a feasibility study.

8a) Method used to generate the random allocation sequence

NPT: When applicable, how care providers were allocated to each trial group

Does your paper address CONSORT subitem 8a? *

Copy and paste relevant sections from the manuscript (include quotes in quotation marks "like this" to indicate direct quotes from your manuscript), or elaborate on this item by providing additional information not in the ms, or briefly explain why the item is not applicable/relevant for your study

"All participants who provided informed consent and completed the T0 questionnaires were randomised into the IG or CG using a 1:1 ratio, via the randomisation function within the Qualtrics Survey Software"

8b) Type of randomisation; details of any restriction (such as blocking and block size)

Does your paper address CONSORT subitem 8b? *

Copy and paste relevant sections from the manuscript (include quotes in quotation marks "like this" to indicate direct quotes from your manuscript), or elaborate on this item by providing additional information not in the ms, or briefly explain why the item is not applicable/relevant for your study

Simple randomisation on a 1:1 ratio

9) Mechanism used to implement the random allocation sequence (such as sequentially numbered containers), describing any steps taken to conceal the sequence until interventions were assigned

Does your paper address CONSORT subitem 9? *

Copy and paste relevant sections from the manuscript (include quotes in quotation marks "like this" to indicate direct quotes from your manuscript), or elaborate on this item by providing additional information not in the ms, or briefly explain why the item is not applicable/relevant for your study

"Participants were informed upon completion of the T0 questionnaires, via a notification in Qualtrics, whether they had been randomised to the intervention group (in this case starting in May 2020), or the control group (in this case, starting in June 2020). The research team remained unaware of participant allocation until group contact lists were created at the next data collection point (i.e. T1)"

10) Who generated the random allocation sequence, who enrolled participants, and who assigned participants to interventions

Does your paper address CONSORT subitem 10? *

Copy and paste relevant sections from the manuscript (include quotes in quotation marks "like this" to indicate direct quotes from your manuscript), or elaborate on this item by providing additional information not in the ms, or briefly explain why the item is not applicable/relevant for your study

"Participants were allocated to the IG or CG via the randomisation function in Qualtrics. Participants were then emailed with a link to the HOPE Program starting on the following week (IG), or a message to say that they would be emailed a link to the HOPE Program (CG) in approximately six weeks' time"

H4C were responsible for emailing the Program link and joining instructions to participants.

11a) If done, who was blinded after assignment to interventions (for example, participants, care providers, those assessing outcomes) and how

NPT: Whether or not administering co-interventions were blinded to group assignment

11a-i) Specify who was blinded, and who wasn't

Specify who was blinded, and who wasn't. Usually, in web-based trials it is not possible to blind the participants [1, 3] (this should be clearly acknowledged), but it may be possible to blind outcome assessors, those doing data analysis or those administering co-interventions (if any).

	1	2	3	4	5	
subitem not at all important	○	○	○	○	◉	essential

Clear selection

Does your paper address subitem 11a-i? *

Copy and paste relevant sections from the manuscript (include quotes in quotation marks "like this" to indicate direct quotes from your manuscript), or elaborate on this item by providing additional information not in the ms, or briefly explain why the item is not applicable/relevant for your study

"Owing to the nature of the study design, it was not possible to blind participants to their group allocation. However, statistical analyses of study data were conducted blind to participant allocation where possible (e.g. IG and CG were labelled 'A' and 'B', arbitrarily)"

11a-ii) Discuss e.g., whether participants knew which intervention was the “intervention of interest” and which one was the “comparator”

Informed consent procedures (4a-ii) can create biases and certain expectations - discuss e.g., whether participants knew which intervention was the “intervention of interest” and which one was the “comparator”.

	1	2	3	4	5	
subitem not at all important	○	○	○	◉	○	essential

Clear selection

Does your paper address subitem 11a-ii?

Copy and paste relevant sections from the manuscript (include quotes in quotation marks "like this" to indicate direct quotes from your manuscript), or elaborate on this item by providing additional information not in the ms, or briefly explain why the item is not applicable/relevant for your study

"All participants received access to the same digital HOPE Program. The IG received access immediately, and the CG were granted access approximately 6 weeks later"

Therefore, participants knew that they would receive access to the HOPE Program - either immediately or after a 6 week wait (waitlist control).

11b) If relevant, description of the similarity of interventions

(this item is usually not relevant for ehealth trials as it refers to similarity of a placebo or sham intervention to a active medication/intervention)

Does your paper address CONSORT subitem 11b? *

Copy and paste relevant sections from the manuscript (include quotes in quotation marks "like this" to indicate direct quotes from your manuscript), or elaborate on this item by providing additional information not in the ms, or briefly explain why the item is not applicable/relevant for your study

Not applicable - waitlist control group had access to the intervention after the waiting period.

12a) Statistical methods used to compare groups for primary and secondary outcomes

NPT: When applicable, details of whether and how the clustering by care providers or centers was addressed

Does your paper address CONSORT subitem 12a? *

Copy and paste relevant sections from the manuscript (include quotes in quotation marks "like this" to indicate direct quotes from your manuscript), or elaborate on this item by providing additional information not in the ms, or briefly explain why the item is not applicable/relevant for your study

"Quantitative data were analysed descriptively using IBM SPSS Statistics 26 (IBM Corp. Released 2019. IBM SPSS Statistics for Windows, Version 26.0. Armonk, NY: IBM Corp.). Initial analyses involved tabulated and graphical summaries of primary and secondary outcomes for each randomised group using means and variance, including confidence intervals and standard deviations, and number and percentages for categorical variables, to describe the full range of data at baseline and postprogram. An intention to treat (ITT) analysis was carried out, where missing data were rectified using the last observation carried forward [45]. In line with CONSORT guidelines [46], a per protocol analysis was also performed on secondary outcome data from intervention completers, and is reported in the Ancillary Analyses section below.

The study was not powered to perform inferential statistical analyses, and so to signal efficacy, we report pre- and postprogram mean differences and confidence intervals for scores on key secondary outcome measures for the IG and CG"

12a-i) Imputation techniques to deal with attrition / missing values

Imputation techniques to deal with attrition / missing values: Not all participants will use the intervention/comparator as intended and attrition is typically high in ehealth trials. Specify how participants who did not use the application or dropped out from the trial were treated in the statistical analysis (a complete case analysis is strongly discouraged, and simple imputation techniques such as LOCF may also be problematic [4]).

	1	2	3	4	5	
subitem not at all important	○	○	○	○	◉	essential

Clear selection

Does your paper address subitem 12a-i? *

Copy and paste relevant sections from the manuscript (include quotes in quotation marks "like this" to indicate direct quotes from your manuscript), or elaborate on this item by providing additional information not in the ms, or briefly explain why the item is not applicable/relevant for your study

"An intention to treat (ITT) analysis was carried out, where missing data were rectified using the last observation carried forward [45]"

12b) Methods for additional analyses, such as subgroup analyses and adjusted analyses

Does your paper address CONSORT subitem 12b? *

Copy and paste relevant sections from the manuscript (include quotes in quotation marks "like this" to indicate direct quotes from your manuscript), or elaborate on this item by providing additional information not in the ms, or briefly explain why the item is not applicable/relevant for your study

"In line with CONSORT guidelines [46], a per protocol analysis was also performed on secondary outcome data from intervention completers, and is reported in the Ancillary Analyses section below"

X26) REB/IRB Approval and Ethical Considerations [recommended as subheading under "Methods"] (not a CONSORT item)

X26-i) Comment on ethics committee approval

	1	2	3	4	5	
subitem not at all important	○	○	○	○	◉	essential

Clear selection

Does your paper address subitem X26-i?

Copy and paste relevant sections from the manuscript (include quotes in quotation marks "like this" to indicate direct quotes from your manuscript), or elaborate on this item by providing additional information not in the ms, or briefly explain why the item is not applicable/relevant for your study

"The research study was approved by Coventry University Ethics Committee (P106024) on April 28, 2020"

x26-ii) Outline informed consent procedures

Outline informed consent procedures e.g., if consent was obtained offline or online (how? Checkbox, etc.?), and what information was provided (see 4a-ii). See [6] for some items to be included in informed consent documents.

	1	2	3	4	5	
subitem not at all important	○	○	○	○	◉	essential

Clear selection

Does your paper address subitem X26-ii?

Copy and paste relevant sections from the manuscript (include quotes in quotation marks "like this" to indicate direct quotes from your manuscript), or elaborate on this item by providing additional information not in the ms, or briefly explain why the item is not applicable/relevant for your study

Consent was obtained online via Qualtrics. Participants opted in by agreeing to statements of consent as follows:

1. I confirm that I have read and understood the Information Sheet for the Hope study, and that I have had the opportunity to ask any questions

2. I understand that you will be collecting the following data from me: basic sociodemographic information e.g. postcode, age, gender, basic information about my condition, mental health and wellbeing measures (these will be collected using separate measures)

3. I understand that all the information I provide will be treated in confidence

4. I understand that my participation is voluntary and that I am free to change my mind and withdraw at any time, without giving a reason, and that my data can be deleted up until (31/07/2023) by emailing (a.turner@coventry.ac.uk)

5. I understand that the data from the Hope study may be used in an anonymised form for scientific publications and presentations

6. I agree to take part in the study

The informed consent form is published in the Protocol for this trial (see Multimedia Appendix 2), which can be accessed here: https://www.researchprotocols.org/2020/12/e24264

X26-iii) Safety and security procedures

Safety and security procedures, incl. privacy considerations, and any steps taken to reduce the likelihood or detection of harm (e.g., education and training, availability of a hotline)

	1	2	3	4	5	
subitem not at all important	○	○	○	○	●	essential

Clear selection

Does your paper address subitem X26-iii?

Copy and paste relevant sections from the manuscript (include quotes in quotation marks "like this" to indicate direct quotes from your manuscript), or elaborate on this item by providing additional information not in the ms, or briefly explain why the item is not applicable/relevant for your study

"In line with the trial protocol [24], participants who indicated self-harm or suicidal thoughts on the PHQ-9 measure were contacted, along with the MCS administrator, by H4C and were provided with the contact details of local mental health agencies, Samaritans, and encouraged to visit their GP"

"...participants scoring [illegible]10 on the PHQ9, or [illegible]8 on the GAD7, were categorized as having reached a probable clinical level of depression or anxiety, respectively...all of these participants were contacted by H4C and encouraged to visit their GP and were signposted to further sources of support as listed above"

RESULTS

13a) For each group, the numbers of participants who were randomly assigned, received intended treatment, and were analysed for the primary outcome

NPT: The number of care providers or centers performing the intervention in each group and the number of patients treated by each care provider in each center

Does your paper address CONSORT subitem 13a? *

Copy and paste relevant sections from the manuscript (include quotes in quotation marks "like this" to indicate direct quotes from your manuscript), or elaborate on this item by providing additional information not in the ms, or briefly explain why the item is not applicable/relevant for your study

"The total number of participants enrolled in the study was n=41, with n=21 in the IG and n=20 in the CG. All participants completed baseline (T0) questionnaires, and missing data in T1 and T2 questionnaires was populated with the last observation carried forward (LOCF) method for the intention to treat analysis (ITT). Therefore, the entire sample was included in the ITT analysis (ITT whole sample n=41; IG n=21, CG n=20). Numbers for the per protocol analysis are details in the Ancillary Analyses section below"

"Ancillary analyses
We conducted a per protocol (PP) analysis, which included only those participants who completed all study questionnaires and attended at least three intervention sessions (PP whole sample n=25; IG n=13, CG n=12)"

13b) For each group, losses and exclusions after randomisation, together with reasons

Does your paper address CONSORT subitem 13b? (NOTE: Preferably, this is shown in a CONSORT flow diagram) *

Copy and paste relevant sections from the manuscript (include quotes in quotation marks "like this" to indicate direct quotes from your manuscript), or elaborate on this item by providing additional information not in the ms, or briefly explain why the item is not applicable/relevant for your study

A CONSORT flow diagram is provided in the manuscript.

13b-i) Attrition diagram

Strongly recommended: An attrition diagram (e.g., proportion of participants still logging in or using the intervention/comparator in each group plotted over time, similar to a survival curve) or other figures or tables demonstrating usage/dose/engagement.

	1	2	3	4	5	
subitem not at all important	○	○	○	●	○	essential

Clear selection

Does your paper address subitem 13b-i?

Copy and paste relevant sections from the manuscript or cite the figure number if applicable (include quotes in quotation marks "like this" to indicate direct quotes from your manuscript), or elaborate on this item by providing additional information not in the ms, or briefly explain why the item is not applicable/relevant for your study

Please see Tables 3 and 4 in the manuscript for details of adherence and engagement.

14a) Dates defining the periods of recruitment and follow-up

Does your paper address CONSORT subitem 14a? *

Copy and paste relevant sections from the manuscript (include quotes in quotation marks "like this" to indicate direct quotes from your manuscript), or elaborate on this item by providing additional information not in the ms, or briefly explain why the item is not applicable/relevant for your study

"Recruitment started on April 30, 2020, and ended on May 5, 2020. Data collection started on April 30, 2020, for T0 baseline questionnaires and finished on September 2, 2020, for T2 follow up questionnaires (CG only), which was four weeks after the end of the intervention for the CG as specified in the trial protocol"

14a-i) Indicate if critical “secular events” fell into the study period

Indicate if critical “secular events” fell into the study period, e.g., significant changes in Internet resources available or “changes in computer hardware or Internet delivery resources”

	1	2	3	4	5	
subitem not at all important	○	◉	○	○	○	essential

Clear selection

Does your paper address subitem 14a-i?

Copy and paste relevant sections from the manuscript (include quotes in quotation marks "like this" to indicate direct quotes from your manuscript), or elaborate on this item by providing additional information not in the ms, or briefly explain why the item is not applicable/relevant for your study

There were no secular events to report.

14b) Why the trial ended or was stopped (early)

Does your paper address CONSORT subitem 14b? *

Copy and paste relevant sections from the manuscript (include quotes in quotation marks "like this" to indicate direct quotes from your manuscript), or elaborate on this item by providing additional information not in the ms, or briefly explain why the item is not applicable/relevant for your study

The trial continued to the end, as planned.

15) A table showing baseline demographic and clinical characteristics for each group

NPT: When applicable, a description of care providers (case volume, qualification, expertise, etc.) and centers (volume) in each group

Does your paper address CONSORT subitem 15? *

Copy and paste relevant sections from the manuscript (include quotes in quotation marks "like this" to indicate direct quotes from your manuscript), or elaborate on this item by providing additional information not in the ms, or briefly explain why the item is not applicable/relevant for your study

"Table 2. Baseline characteristics for the whole sample, and by trial arm"

15-i) Report demographics associated with digital divide issues

In ehealth trials it is particularly important to report demographics associated with digital divide issues, such as age, education, gender, social-economic status, computer/Internet/ehealth literacy of the participants, if known.

	1	2	3	4	5	
subitem not at all important	○	○	○	◉	○	essential

Clear selection

Does your paper address subitem 15-i? *

Copy and paste relevant sections from the manuscript (include quotes in quotation marks "like this" to indicate direct quotes from your manuscript), or elaborate on this item by providing additional information not in the ms, or briefly explain why the item is not applicable/relevant for your study

Please see Table 2.

"Sociodemographic and health information collected at baseline (T0) for the whole group, and by treatment group, are presented in Table 2. The sample consisted mostly of white (n=36/41, 87.8%) females (n=32/41, 78.0%) with an average age of 54.3 years. Over half of the sample (n=24/41, 58.5%) had post-school qualifications. The majority of participants were married or living with their partner (n=30/41, 73.2%), and just over half were employed (n=21/41, 51.2%), with just under half reporting that they had to reduce their working hours due to their cancer diagnosis (n=20/41, 48.8%). This variable was the most disproportionate across the trial arms, with over twice as many reports of cutting work hours in the IG (n=14/21, 66.7%) than in the CG (n=6/20, 30.0%)"

16) For each group, number of participants (denominator) included in each analysis and whether the analysis was by original assigned groups

16-i) Report multiple “denominators” and provide definitions

Report multiple “denominators” and provide definitions: Report N’s (and effect sizes) “across a range of study participation [and use] thresholds” [1], e.g., N exposed, N consented, N used more than x times, N used more than y weeks, N participants “used” the intervention/comparator at specific pre-defined time points of interest (in absolute and relative numbers per group). Always clearly define “use” of the intervention.

	1	2	3	4	5	
subitem not at all important	○	○	○	○	◉	essential

Clear selection

Does your paper address subitem 16-i? *

Copy and paste relevant sections from the manuscript (include quotes in quotation marks "like this" to indicate direct quotes from your manuscript), or elaborate on this item by providing additional information not in the ms, or briefly explain why the item is not applicable/relevant for your study

"Over three quarters of the participants invited (N=47/61, 77.0%) were willing to provide consent and be randomised to either the HOPE Program starting the following week, or to a 6-week waiting list. Just over half of the sample (i.e., IG and CG combined; n=21/41, 51.2%) completed all 6 sessions of the intervention, and almost two-thirds of the sample (n=26/41, 63.4%) completed at least 3 sessions (note that n=1 did not complete the T1 questionnaire so was not categorized as an intervention completer – see below). The follow up rate was encouraging, with a large proportion of participants completing the study questionnaires at T1 (n=33/41, 80.5%). Twenty-five of these participants who completed T1 questionnaires also attended [illegible]3 intervention sessions, meeting the criteria for intervention completion (n=25/41, 61.0%). In terms of engagement, within the sessions participants viewed between half and three quarters of the content, on average (range 76.1% – 51.5%)"

Please also see Tables 3-7 in the manuscript.

16-ii) Primary analysis should be intent-to-treat

Primary analysis should be intent-to-treat, secondary analyses could include comparing only “users”, with the appropriate caveats that this is no longer a randomized sample (see 18-i).

	1	2	3	4	5	
subitem not at all important	○	○	○	○	◉	essential

Clear selection

Does your paper address subitem 16-ii?

Copy and paste relevant sections from the manuscript (include quotes in quotation marks "like this" to indicate direct quotes from your manuscript), or elaborate on this item by providing additional information not in the ms, or briefly explain why the item is not applicable/relevant for your study

"An intention to treat (ITT) analysis was carried out, where missing data were rectified using the last observation carried forward [45]"

17a) For each primary and secondary outcome, results for each group, and the estimated effect size and its precision (such as 95% confidence interval)

Does your paper address CONSORT subitem 17a? *

Copy and paste relevant sections from the manuscript (include quotes in quotation marks "like this" to indicate direct quotes from your manuscript), or elaborate on this item by providing additional information not in the ms, or briefly explain why the item is not applicable/relevant for your study

Effect sizes were not reported for this feasibility study. Outcomes are reported as n and percentages, mean scores with standard deviations, mean differences with 95% CIs. Please refer to Tables 3-7 in the manuscript.

17a-i) Presentation of process outcomes such as metrics of use and intensity of use

In addition to primary/secondary (clinical) outcomes, the presentation of process outcomes such as metrics of use and intensity of use (dose, exposure) and their operational definitions is critical. This does not only refer to metrics of attrition (13-b) (often a binary variable), but also to more continuous exposure metrics such as “average session length”. These must be accompanied by a technical description how a metric like a “session” is defined (e.g., timeout after idle time) [1] (report under item 6a).

	1	2	3	4	5	
subitem not at all important	○	○	○	○	◉	essential

Clear selection

Does your paper address subitem 17a-i?

Copy and paste relevant sections from the manuscript (include quotes in quotation marks "like this" to indicate direct quotes from your manuscript), or elaborate on this item by providing additional information not in the ms, or briefly explain why the item is not applicable/relevant for your study

"The intervention platform collects user engagement data such as number of pages viewed in each session and number of goals set which assists the moderators with participant engagement and experience. We measured the mean percentage of pages viewed per session, and number of posts/comments a participant made for key activities (gratitude, setting goals, goal feedback, liking posts, and comments posted)"

"Table 4 shows a selection of engagement data collected by the intervention platform. The mean number of pages viewed per session generally decreased as the program progressed, and ranged from n=12.2/16 (76.3%) in session 1, to n=5.1/10 (51.0%) in session 6, across the whole group. The mean pages viewed in each session was consistent across the whole group and both trial arms for sessions 1 and 6. The mean pages viewed was slightly higher for the CG than the IG for sessions 2-5. Further, the CG tended to set slightly more goals, give more likes, and post more comments than the IG, on average across the course of intervention. There was a negligible difference in mean gratitude entries and goal feedback given between the two trial arms"

17b) For binary outcomes, presentation of both absolute and relative effect sizes is recommended

Does your paper address CONSORT subitem 17b? *

Copy and paste relevant sections from the manuscript (include quotes in quotation marks "like this" to indicate direct quotes from your manuscript), or elaborate on this item by providing additional information not in the ms, or briefly explain why the item is not applicable/relevant for your study

Not applicable for this study.

18) Results of any other analyses performed, including subgroup analyses and adjusted analyses, distinguishing pre-specified from exploratory

Does your paper address CONSORT subitem 18? *

Copy and paste relevant sections from the manuscript (include quotes in quotation marks "like this" to indicate direct quotes from your manuscript), or elaborate on this item by providing additional information not in the ms, or briefly explain why the item is not applicable/relevant for your study

"We conducted a per protocol (PP) analysis, which included only those participants who completed all study questionnaires and attended at least three intervention sessions (PP whole sample n=25; IG n=13, CG n=12). Table 7 shows the data from the secondary outcome measures for these participants. The data in Table 7 show similar patterns to those of the ITT in Table 5 above. On average, participants in the IG made modest improvements from T0 to T1. Participants in the CG showed little to no improvements in the same period. The PP analysis shows difference in change scores (final column) of greater magnitude comparative to the ITT analysis"

18-i) Subgroup analysis of comparing only users

A subgroup analysis of comparing only users is not uncommon in ehealth trials, but if done, it must be stressed that this is a self-selected sample and no longer an unbiased sample from a randomized trial (see 16-iii).

	1	2	3	4	5	
subitem not at all important	○	○	○	◉	○	essential

Clear selection

Does your paper address subitem 18-i?

Copy and paste relevant sections from the manuscript (include quotes in quotation marks "like this" to indicate direct quotes from your manuscript), or elaborate on this item by providing additional information not in the ms, or briefly explain why the item is not applicable/relevant for your study

As above.

"We conducted a per protocol (PP) analysis, which included only those participants who completed all study questionnaires and attended at least three intervention sessions (PP whole sample n=25; IG n=13, CG n=12). Table 7 shows the data from the secondary outcome measures for these participants. The data in Table 7 show similar patterns to those of the ITT in Table 5 above. On average, participants in the IG made modest improvements from T0 to T1. Participants in the CG showed little to no improvements in the same period. The PP analysis shows difference in change scores (final column) of greater magnitude comparative to the ITT analysis"

19) All important harms or unintended effects in each group

(for specific guidance see CONSORT for harms)

Does your paper address CONSORT subitem 19? *

Copy and paste relevant sections from the manuscript (include quotes in quotation marks "like this" to indicate direct quotes from your manuscript), or elaborate on this item by providing additional information not in the ms, or briefly explain why the item is not applicable/relevant for your study

"Harms
In line with the trial protocol [24], participants who indicated self-harm or suicidal thoughts on the PHQ-9 measure were contacted, along with the MCS administrator, by H4C and were provided with the contact details of local mental health agencies, Samaritans, and encouraged to visit their GP. This was the case for 22.0% (n=9/41) of participants preprogram, and 9.8% (n=4/41) of participants postprogram (data not shown in Tables). At postprogram, there were no participants who indicated self-harm or suicidal thoughts where they had not already indicated this at preprogram.

As detailed in the Methods section, participants scoring [illegible]10 on the PHQ9, or [illegible]8 on the GAD7, were categorized as having reached a probable clinical level of depression or anxiety, respectively. Depression was indicated in 43.9% (n=18/41) of participants at preprogram, and 34.1% (n=14/41) at T1. Anxiety was indicated in 48.8% (n=20/41) of participants at preprogram, and 43.9% (n=18/41) at T1. In line with the trial protocol [24], all of these participants were contacted by H4C and encouraged to visit their GP and were signposted to further sources of support as listed above.

At postprogram, there were no participants who reported a probable clinical level of depression where they had not already reported this at preprogram. However, at postprogram, 4.9% (n=2/41) of participants reached a probable clinical level of anxiety but were not previously at this level at preprogram. Both participants scored 7 on the GAD7 measure at preprogram, increasing to scores of 8 (n=1) and 9 (n=1) at postprogram. Both participants were contacted by H4C, as outlined above and in the trial protocol [24]. To provide further context, both participants were in the IG, and only attended one (n=1) or two (n=1) sessions of the intervention. Both participants were still undergoing treatment for their cancer and one described significant personal stress unrelated to their cancer. Whilst we cannot rule out the possibility that the intervention may have caused the increased anxiety in these two participants, they did not engage in more than two sessions of the intervention and the context of the COVID pandemic is linked to increased anxiety amongst cancer patients [1,3,5,6]. Furthermore, other participants did show positive changes in their pre-postprogram mental wellbeing scores"

19-i) Include privacy breaches, technical problems

Include privacy breaches, technical problems. This does not only include physical “harm” to participants, but also incidents such as perceived or real privacy breaches [1], technical problems, and other unexpected/unintended incidents. “Unintended effects” also includes unintended positive effects [2].

	1	2	3	4	5	
subitem not at all important	○	○	○	●	○	essential

Clear selection

Does your paper address subitem 19-i?

Copy and paste relevant sections from the manuscript (include quotes in quotation marks "like this" to indicate direct quotes from your manuscript), or elaborate on this item by providing additional information not in the ms, or briefly explain why the item is not applicable/relevant for your study

No privacy breaches or technical problems to report.

19-ii) Include qualitative feedback from participants or observations from staff/researchers

Include qualitative feedback from participants or observations from staff/researchers, if available, on strengths and shortcomings of the application, especially if they point to unintended/unexpected effects or uses. This includes (if available) reasons for why people did or did not use the application as intended by the developers.

	1	2	3	4	5	
subitem not at all important	○	○	●	○	○	essential

Clear selection

Does your paper address subitem 19-ii?

Copy and paste relevant sections from the manuscript (include quotes in quotation marks "like this" to indicate direct quotes from your manuscript), or elaborate on this item by providing additional information not in the ms, or briefly explain why the item is not applicable/relevant for your study

Not included on the advice of a peer reviewer.

DISCUSSION

22) Interpretation consistent with results, balancing benefits and harms, and considering other relevant evidence

NPT: In addition, take into account the choice of the comparator, lack of or partial blinding, and unequal expertise of care providers or centers in each group

22-i) Restate study questions and summarize the answers suggested by the data, starting with primary outcomes and process outcomes (use)

Restate study questions and summarize the answers suggested by the data, starting with primary outcomes and process outcomes (use).

	1	2	3	4	5	
subitem not at all important	○	○	○	○	◉	essential

Clear selection

Does your paper address subitem 22-i? *

Copy and paste relevant sections from the manuscript (include quotes in quotation marks "like this" to indicate direct quotes from your manuscript), or elaborate on this item by providing additional information not in the ms, or briefly explain why the item is not applicable/relevant for your study

"The feasibility RCT of the digital HOPE Program aimed to assess primary outcomes measuring trial feasibility, and secondary outcomes relating to measures of participant wellbeing. The trial yielded encouraging data on the primary outcome measures of recruitment, retention, follow up, adherence and engagement rates"

22-ii) Highlight unanswered new questions, suggest future research

Highlight unanswered new questions, suggest future research.

	1	2	3	4	5	
subitem not at all important	○	○	○	○	◉	essential

Clear selection

Does your paper address subitem 22-ii?

Copy and paste relevant sections from the manuscript (include quotes in quotation marks "like this" to indicate direct quotes from your manuscript), or elaborate on this item by providing additional information not in the ms, or briefly explain why the item is not applicable/relevant for your study

"This feasibility RCT was not powered to detect statistically significant differences in pre-post scores on secondary outcomes. However, the results indicate that the HOPE Program has the potential to have a positive effect on mental wellbeing, depression and anxiety in people with cancer. These have been identified as important outcomes for people with cancer [9-11] and echo the results of a previous pre-post study of the HOPE Program [23], giving further confidence in the potential efficacy of the intervention. Data from a fully powered trial will allow us, for the first time, to report statistically significant differences in pre- and postprogram scores for both an intervention and a control group. However, unless we account for expectancy effects, we cannot be sure about the efficacy of the intervention [48]. Therefore, future trials will need to employ an appropriate active control program, which equates expectations to those of the intervention group, to allow a causal conclusion about the effectiveness of the intervention effectiveness"

20) Trial limitations, addressing sources of potential bias, imprecision, and, if relevant, multiplicity of analyses

20-i) Typical limitations in ehealth trials

Typical limitations in ehealth trials: Participants in ehealth trials are rarely blinded. Ehealth trials often look at a multiplicity of outcomes, increasing risk for a Type I error. Discuss biases due to non-use of the intervention/usability issues, biases through informed consent procedures, unexpected events.

	1	2	3	4	5	
subitem not at all important	○	◉	○	○	○	essential

Clear selection

Does your paper address subitem 20-i? *

Copy and paste relevant sections from the manuscript (include quotes in quotation marks "like this" to indicate direct quotes from your manuscript), or elaborate on this item by providing additional information not in the ms, or briefly explain why the item is not applicable/relevant for your study

"This study found that overall engagement measured by the percentage of pages viewed seemed to decline as participants progressed through the sessions. This may be due to fatigue, or redundant content, for example. Qualitative investigation into what content participants engaged with, and elements they found more or less relevant/helpful, would be a useful supplement to improve the intervention"

21) Generalisability (external validity, applicability) of the trial findings

NPT: External validity of the trial findings according to the intervention, comparators, patients, and care providers or centers involved in the trial

21-i) Generalizability to other populations

Generalizability to other populations: In particular, discuss generalizability to a general Internet population, outside of a RCT setting, and general patient population, including applicability of the study results for other organizations

	1	2	3	4	5	
subitem not at all important	○	○	○	○	◉	essential

Clear selection

Does your paper address subitem 21-i?

Copy and paste relevant sections from the manuscript (include quotes in quotation marks "like this" to indicate direct quotes from your manuscript), or elaborate on this item by providing additional information not in the ms, or briefly explain why the item is not applicable/relevant for your study

"The recruitment for this feasibility RCT was from an opportunity sample of self-selecting participants referred by Macmillan Cancer Support. The self-selecting nature of the recruitment strategy may yield participants who are generally more motivated to seek help and/or help themselves. However, this recruitment strategy facilitated the rapid attainment of trial recruitment targets for this study [49]. Research has shown that recruitment via social media is more effective if advertised by a collaborating cancer charity [50]. In this respect, in the current climate of increased need for digital research and provision of self-management support, we have optimised our recruitment strategy and would adopt this again in a definitive trial.

The majority of the participants were white (n=36, 87.8%), female (n=32, 78.0%), married (n=30, 73.2), and educated (n=24, 58.5%), and the most commonly reported type of cancer was breast cancer (n=17, 41.5%). This likely relates to the demographics of people who engage with Macmillan Cancer Support charity. Although this may limit the generalizability of the results to other demographic groups, some aspects are in line with wider population statistics and research findings. The 2011 Census [51] reported that 86.0% of the population in England and Wales were white, so the sample in this study is representative of the wider population in this respect. Breast cancer is the most common type of cancer in the UK, accounting for 15.1% of malignant cancer registrations in England in 2017 [52], yet breast cancer was reported by 41.5% of participants in this study. The data presented in this study may not be representative of other cancer populations. As such, the efficacy signal and feasibility findings of this study may not be generalisable to other types of cancer, or to non-white, males for example. We will seek advice from our partners and trial experts before proceeding to a definitive trial. It may be more appropriate to run a definitive RCT of the HOPE Program for breast cancer survivors only, since i) breast cancer is the most commonly diagnosed cancer in the UK, and ii) our own data [e.g. 23 and unpublished studies] show that it is mainly women with breast cancer who take part in the HOPE Program. However, the HOPE Program was designed to help people living with all types of cancer, and so the community HOPE Program run by MCS will continue to be open to all cancer survivors.

A low attendance rate for men is common in self-management and is linked to their reluctance to seek help [53]. In terms of recruitment, men are more likely to respond to marketing and recruitment messages that emphasize stoicism, independence, and control [53] and where the materials contain images of men [54]. Once recruited, there are also qualitative differences in how men and women engage with their peers in same- or mixed-sex online cancer support groups [55]. A recent systematic review confirms that men are more oriented towards informational support, and women towards emotional support [56]. In terms of the current intervention, further intervention development is required to ensure relevance and acceptability of the intervention, and potentially to co-design tailored versions for more diverse groups and communities. This may require, i) further consultation with MCS to co-design specific programs for gendered cancers (e.g. a HOPE Program for testicular, prostrate, or breast cancer), ii) co-development of course content and recruitment materials to increase the engagement of men in a general cancer intervention, iii) partnering with different charities to enhance engagement with people with cancer from different

ethnic groups, socio-economic status and educational attainment. A future trial could examine the feasibility of recruitment through the NHS, from clinics, consultation rooms or waiting rooms, to broaden the recruitment strategy to wider communities. For future cohorts, we will encourage MCS to review their recruitment materials to ensure that they contain images and messages that appeal to multiple audiences, and are advertised in (largely online) areas and locations frequented by people of all ages, ethnicities, genders, and income groups [53-56]"

21-ii) Discuss if there were elements in the RCT that would be different in a routine application setting

Discuss if there were elements in the RCT that would be different in a routine application setting (e.g., prompts/reminders, more human involvement, training sessions or other co-interventions) and what impact the omission of these elements could have on use, adoption, or outcomes if the intervention is applied outside of a RCT setting.

	1	2	3	4	5	
subitem not at all important	○	○	○	●	○	essential

Clear selection

Does your paper address subitem 21-ii?

Copy and paste relevant sections from the manuscript (include quotes in quotation marks "like this" to indicate direct quotes from your manuscript), or elaborate on this item by providing additional information not in the ms, or briefly explain why the item is not applicable/relevant for your study

Not applicable to this study.

OTHER INFORMATION

23) Registration number and name of trial registry

Does your paper address CONSORT subitem 23? *

Copy and paste relevant sections from the manuscript (include quotes in quotation marks "like this" to indicate direct quotes from your manuscript), or elaborate on this item by providing additional information not in the ms, or briefly explain why the item is not applicable/relevant for your study

"This feasibility randomised wait-list control trial was retrospectively registered with the ISRCTN registry (https://www.isrctn.com/ISRCTN79623250) on Nov 4, 2020"

24) Where the full trial protocol can be accessed, if available

Does your paper address CONSORT subitem 24? *

Cite a Multimedia Appendix, other reference, or copy and paste relevant sections from the manuscript (include quotes in quotation marks "like this" to indicate direct quotes from your manuscript), or elaborate on this item by providing additional information not in the ms, or briefly explain why the item is not applicable/relevant for your study

"The feasibility trial protocol has been registered and published [International Registered Report Identifier (IRRID): DERR1-10.2196/24264 [24]"

25) Sources of funding and other support (such as supply of drugs), role of funders

Does your paper address CONSORT subitem 25? *

Copy and paste relevant sections from the manuscript (include quotes in quotation marks "like this" to indicate direct quotes from your manuscript), or elaborate on this item by providing additional information not in the ms, or briefly explain why the item is not applicable/relevant for your study

"The research study was investigator funded and was approved by Coventry University Ethics Committee (P106024) on April 28, 2020"

X27) Conflicts of Interest (not a CONSORT item)

X27-i) State the relation of the study team towards the system being evaluated

In addition to the usual declaration of interests (financial or otherwise), also state the relation of the study team towards the system being evaluated, i.e., state if the authors/evaluators are distinct from or identical with the developers/sponsors of the intervention.

	1	2	3	4	5	
subitem not at all important	○	○	○	◉	○	essential

Clear selection

Does your paper address subitem X27-i?

Copy and paste relevant sections from the manuscript (include quotes in quotation marks "like this" to indicate direct quotes from your manuscript), or elaborate on this item by providing additional information not in the ms, or briefly explain why the item is not applicable/relevant for your study

Andy Turner is co-founder of the HOPE Program and non-executive director of H4C.
Gabriela Matouskova is CEO of H4C.

About the CONSORT EHEALTH checklist

As a result of using this checklist, did you make changes in your manuscript? *

- ○ yes, major changes
- ○ yes, minor changes
- ◉ no

What were the most important changes you made as a result of using this checklist?

Your answer

How much time did you spend on going through the checklist INCLUDING making changes in your manuscript *

Difficult to quantify, as I had to complete it in stages, but likely around 4 hours.

As a result of using this checklist, do you think your manuscript has improved? *

- ○ yes
- ◉ no
- ○ Other:

Would you like to become involved in the CONSORT EHEALTH group?

This would involve for example becoming involved in participating in a workshop and writing an "Explanation and Elaboration" document

- ○ yes
- ◉ no
- ○ Other:

Clear selection

Any other comments or questions on CONSORT EHEALTH

It takes far too long to complete the checklist - especially after out manuscript has been accepted and no changes can be made now anyway. A lot of the information provided on the checklist was duplication of what is presented in the paper - which was prepared in line with the CONSORT guide for reporting clinical trials. I'm not clear what this checklist adds.

STOP - Save this form as PDF before you click submit

To generate a record that you filled in this form, we recommend to generate a PDF of this page (on a Mac, simply select "print" and then select "print as PDF") before you submit it.

When you submit your (revised) paper to JMIR, please upload the PDF as supplementary file.

Don't worry if some text in the textboxes is cut off, as we still have the complete information in our database. Thank you!

Final step: Click submit !

Click submit so we have your answers in our database!

Submit

Clear form

Never submit passwords through Google Forms.

This content is neither created nor endorsed by Google. Report Abuse - Terms of Service - Privacy Policy

Google Forms
